# Differential microRNA Expression in Fast- and Slow-Twitch Skeletal Muscle of *Piaractus mesopotamicus* during Growth

**DOI:** 10.1371/journal.pone.0141967

**Published:** 2015-11-03

**Authors:** Bruno Oliveira da Silva Duran, Geysson Javier Fernandez, Edson Assunção Mareco, Leonardo Nazario Moraes, Rondinelle Artur Simões Salomão, Tassiana Gutierrez de Paula, Vander Bruno Santos, Robson Francisco Carvalho, Maeli Dal-Pai-Silvca

**Affiliations:** 1 Department of Morphology, Institute of Biosciences of Botucatu, São Paulo State University, Botucatu, São Paulo, Brazil; 2 Aquaculture Center, São Paulo State University, Jaboticabal, São Paulo, Brazil; 3 São Paulo Agency for Agribusiness Technology, Presidente Prudente, São Paulo, Brazil; Universidad Pablo de Olavide, Centro Andaluz de Biología del Desarrollo-CSIC, SPAIN

## Abstract

Pacu (*Piaractus mesopotamicus*) is a Brazilian fish with a high economic value in pisciculture due to its rusticity and fast growth. Postnatal growth of skeletal muscle in fish occurs by hyperplasia and/or hypertrophy, processes that are dependent on the proliferation and differentiation of myoblasts. A class of small noncoding RNAs, known as microRNAs (miRNAs), represses the expression of target mRNAs, and many studies have demonstrated that miR-1, miR-133, miR-206 and miR-499 regulate different processes in skeletal muscle through the mRNA silencing of *hdac4* (*histone deacetylase 4*), *srf* (*serum response factor*), *pax7* (*paired box 7*) and *sox6* ((*sex determining region Y)-box 6*), respectively. The aim of our work was to evaluate the expression of these miRNAs and their putative target mRNAs in fast- and slow-twitch skeletal muscle of pacu during growth. We used pacus in three different development stages: larval (aged 30 days), juvenile (aged 90 days and 150 days) and adult (aged 2 years). To complement our study, we also performed a pacu myoblast cell culture, which allowed us to investigate miRNA expression in the progression from myoblast proliferation to differentiation. Our results revealed an inverse correlation between the expression of the miRNAs and their target mRNAs, and there was evidence that miR-1 and miR-206 may regulate the differentiation of myoblasts, whereas miR-133 may regulate the proliferation of these cells. miR-499 was highly expressed in slow-twitch muscle, which suggests its involvement in the specification of the slow phenotype in muscle fibers. The expression of these miRNAs exhibited variations between different development stages and between distinct muscle twitch phenotypes. This work provides the first identification of miRNA expression profiles in pacu skeletal muscle and suggests an important role of these molecules in muscle growth and in the maintenance of the muscle phenotype.

## Introduction

*Piaractus mesopotamicus*, popularly known as pacu, is a fish whose distribution is more concentrated in the wetland areas of the Brazilian Midwest region, and it is one of the most studied fish in this country [1]. This species is characterized by rusticity, fast growth, adaptation to artificial feeding and tasty meat [2], and therefore has a high market value to Brazilian fishery and pisciculture. In addition to its high economic value, pacu is also important in scientific research. Zebrafish (*Danio rerio*) and medaka (*Oryzias latipes*) are widely used as model organisms for the understanding of many biological processes, but there are some limitations due to their small sizes, such as the study of myogenesis in cell cultures [3]. Because pacus have a large body size, they have the potential to be an excellent model for studies of muscle growth in fish.

In most fish, skeletal muscle constitutes ~60% of the body mass and comprises two major fiber types: fast-twitch muscle fibers, which comprise the main edible part of the fish, and slow-twitch muscle fibers [4]. Postnatal muscle growth in fish involves cells localized at the periphery of muscle fibers, known as satellite cells. These cells give rise to myoblasts, the proliferation and differentiation of which are the mechanisms that promote hyperplasia (an increase in muscle fiber number) and hypertrophy (an increase in muscle fiber size) [5,6]. These processes are regulated by several transcription factors, such as myogenic regulatory factors (MRFs). MRFs include MyoD, which regulates the specification and proliferation of myoblasts, and Myogenin, which is involved in the differentiation and fusion of these cells [7].

Small noncoding regulatory RNAs, such as microRNAs (miRNAs), also play an important role in vertebrate muscle development. miRNAs are highly conserved molecules whose primary function is post-transcriptional gene regulation through the translational inhibition and decay of messenger RNAs (mRNAs) [8,9]. This repression occurs through nucleotide base complementation between the seed sequence of the miRNA at 5′ region and the 3′ untranslated region (3'UTR) of the target mRNA. Combinatorial regulation is a common feature; an miRNA may have several target mRNAs and, similarly, a single mRNA can be targeted by different miRNAs [10,11]. Thus, miRNAs orchestrate the regulation of their targets to control signaling pathways and common biological functions. Some miRNAs are specifically or highly expressed in cardiac and/or skeletal muscles and are therefore muscle-specific. miR-1, miR-133, miR-206 and miR-499 are involved in several processes in skeletal muscle, including myogenesis, myoblast proliferation and differentiation, fiber type specification and muscle regeneration [12–16].

Several miRNAs exhibit very similar or even identical nucleotide sequences, and, therefore, are often grouped into families [17]. Typically, these miRNAs participate in the same regulatory pathway, possessing similar roles in the biological tissue. miR-1 and miR-206 belong to the same family of miRNAs and are mainly involved in myoblast differentiation in skeletal muscle through silencing of molecules that repress this process, such as *hdac4* (*histone deacetylase 4*) and *pax7* (*paired box 7*) [12,14,18]. Another molecule involved in muscle growth is *srf* (*serum response factor*), the mRNA of which is silenced by miR-133a and miR-133b, which also belong to the same family of miRNAs and are related to myoblast proliferation [12,18]. In addition to these miRNAs, miR-499 is involved in the specification and maintenance of the slow-twitch phenotype in muscle fibers, particularly by silencing *sox6* ((*sex determining region Y)-box 6*), which participates in the definition of the fast-twitch phenotype [13,19,20].

The fish myoblast cell culture, an *in vitro* model, is becoming a very useful tool to understand the regulation of muscle growth [6]. Myoblast cell culture recapitulates the steps of myogenesis [21] and provides an environment that permits the examination of regulatory molecules under precisely controlled conditions [6,22]. Therefore, the pacu myoblast cell culture is an excellent model to complement our study and understand the miRNA-mediated regulation of muscle growth.

We hypothesized that miRNAs are involved in the regulation of skeletal muscle growth and the twitch phenotypes in pacu. The aim of our work was to evaluate the expression of miR-1, miR-133a, miR-133b, miR-206, and miR-499 and their respective putative targets (*hdac4*, *srf*, *pax7* and *sox6*) in fast- and slow-twitch skeletal muscle of pacu during growth and to provide new information on the molecular mechanisms that regulate muscle development in this species. Studies that aim to clarify the molecular mechanisms involved in pacu muscle growth may contribute to improvements in its intensive farming because these mechanisms contribute to changes in the cellularity of the skeletal muscle and, consequently, are directly related to increased muscle mass and meat quality.

## Materials and Methods

### Ethics statement and experimental design

All experiments and procedures were performed in accordance with the Ethical Principles in Animal Research adopted by the Brazilian College of Animal Experimentation (COBEA). The protocol was approved by the Ethics Committee on Animal Use (protocol number 506) of the Institute of Biosciences of Botucatu, São Paulo State University, Botucatu, São Paulo, Brazil. Pacus were obtained from the São Paulo Agency for Agribusiness Technology (APTA), Presidente Prudente, São Paulo, Brazil. We evaluated different development stages: 30-day-old larvae (1.87 ± 0.44 g—mean ± SD), 90-day-old juveniles (36.73 ± 3.73 g—mean ± SD), 150-day-old juveniles (93.23 ± 10.21 g—mean ± SD) and 2-year-old adults (1713.33 ± 190.96 g—mean ± SD). The fish were farmed at 28°C under a natural photoperiod (12 light:12 dark) in 0.5 m^3^ storage tanks equipped with distinct systems for water circulation. The animals were euthanized with excess benzocaine, at a concentration exceeding 250 mg/L, prior to the collection of the muscle samples. The fast-twitch muscle was collected from the epaxial region near the head, and the slow-twitch muscle was collected near the lateral line. We did not collect the slow-twitch muscle from 30- and 90-day-old pacus because of the difficulty in isolating this tissue in these animals. The samples were frozen in liquid nitrogen and stored at -80°C. Fast-twitch muscles were collected from juvenile pacus weighing 31.75 ± 2.53 g (mean ± SD; n = 10 per cell culture) to establish the primary myoblast cell cultures. The myoblasts were isolated and cultured according to the protocol described by Bower & Johnston (2010) [23].

### Myoblast cell culture

The samples of fast-twitch muscle were mechanically dissociated with scalpels. The fragments were enzymatically digested with 0.2% collagenase type I (Sigma-Aldrich, USA) and 5% trypsin (Sigma-Aldrich, USA), allowing for the release of the muscle cells. The cell suspension was filtered in cell strainers (Corning, USA) to remove the debris and resuspended in DMEM media with 10% fetal bovine serum and 1% antibiotics (Sigma-Aldrich, USA). After the cells were counted in a Neubauer chamber, they were diluted at a concentration of 1×10^6^ cells/mL and plated in wells that were previously treated with poly-L-lysine and laminin (Sigma-Aldrich, USA), which have high affinity for the myoblasts. The myoblasts were incubated at 28°C for 12 days and were studied in three phases of the cell cultures: commitment and early proliferation (2–4 days), late proliferation and early fusion (5–8 days), and late fusion and myotube formation (9–12 days). The media was changed every 2 days, and the morphology of myoblasts was regularly monitored.

### miRNAs and target expression

The total RNA was extracted from the muscle samples and myoblast cell cultures using *TRIzol* ^*®*^ *Reagent* (Life Technologies, USA), according to the manufacturer’s recommendations. The RNA was quantified using a *NanoVue™ Plus* spectrophotometer (GE Healthcare, USA), which also allowed an estimation of the RNA purity by measuring the absorbance at 260 nm (RNA quantity) and 280 nm (protein quantity). Only samples with 260/280 ratio ≥ 1.8 were used. The RNA integrity was evaluated through capillary electrophoresis in the *2100 Bioanalyzer* (Agilent, USA), which provided a RNA integrity number (RIN) based on the *28S* and *18S ribosomal RNAs*. Only samples with an RIN ≥ 7.0 were used. The extracted RNA was treated with *DNase I*, *Amplification Grade* (Life Technologies, USA) to eliminate any possible contaminating genomic DNA from the samples. For miRNA expression, the RNA reverse transcription was performed using the *TaqMan* ^*®*^ *MicroRNA Reverse Transcription kit* (Life Technologies, USA) combined with the *TaqMan* ^*®*^ *MicroRNA Assays* (Life Technologies, USA) according to the manufacturer’s instructions. For mRNA expression, the RNA reverse transcription was performed using the *High Capacity cDNA Archive Kit* (Life Technologies, USA) according to the manufacturer’s guidelines.

The miRNAs and mRNAs expression levels were detected by quantitative real-time PCR (qPCR) using the *QuantStudio™ 12K Flex Real-Time PCR System* (Life Technologies, USA). Each cDNA sample that corresponded to a miRNA was amplified using the *TaqMan* ^*®*^ *Universal PCR Master Mix* (Life Technologies, USA) and the *TaqMan* ^*®*^ *MicroRNA Assays* (Life Technologies, USA), which contains primers and specific probes to miR-1, miR-133a, miR-133b, miR-206 and miR-499 (S1 Table). For the *hdac4*, *srf*, *pax7*, *sox6*, *myod* and *myogenin* mRNAs, the cDNA samples were amplified using the *Fast SYBR* ^*®*^ *Green Master Mix* (Life Technologies, USA) and primers synthesized by Life Technologies (USA), which were designed using *Primer Express 3*.*0*.*1*.^*®*^ (Life Technologies, USA) (S2 Table). The reactions were performed under the following conditions: 95°C for 10 minutes, 40 cycles of denaturation at 95°C for 15 seconds and annealing/extension at 60°C for 1 minute. For the samples amplified with the *Fast SYBR* ^*®*^ *Green Master Mix*, the specificity of each primer set was evaluated by analyzing the dissociation curve at the end of each PCR reaction, which confirmed the presence of a single fluorescence peak. The relative quantification of expression was performed by the 2^-ΔΔCt^ method [24] (S1 File), and the miRNA and mRNA expression levels were normalized to the *U6 snRNA* (*U6 small nuclear RNA*) and *18S rRNA* (*18S ribosomal RNA*), respectively, whose expressions were constant among all samples (S2 File). The data are expressed as the fold changes relative to the following calibrators: 30 days group for the fast-twitch muscle, 150 days group for the slow-twitch muscle, fast-twitch muscle for the comparisons between muscle types, and 2–4 days group for the myoblast cell cultures.

### Statistical analyses

Statistical analyses of the expression data during the growth of fast-twitch muscle and the data from the myoblast cell cultures were performed using a parametric one-way ANOVA test, followed by Tukey’s multiple comparisons test. The unpaired t-test was used for the expression data during the growth of slow-twitch muscle and between the fast- and slow-twitch muscles of the 150-day-old and 2-year-old pacus. Statistical significance was set at 5% (p<0.05) (GraphPad Prism 5 Software, USA).

## Results

### miRNAs and target expression

**Fig 1:**
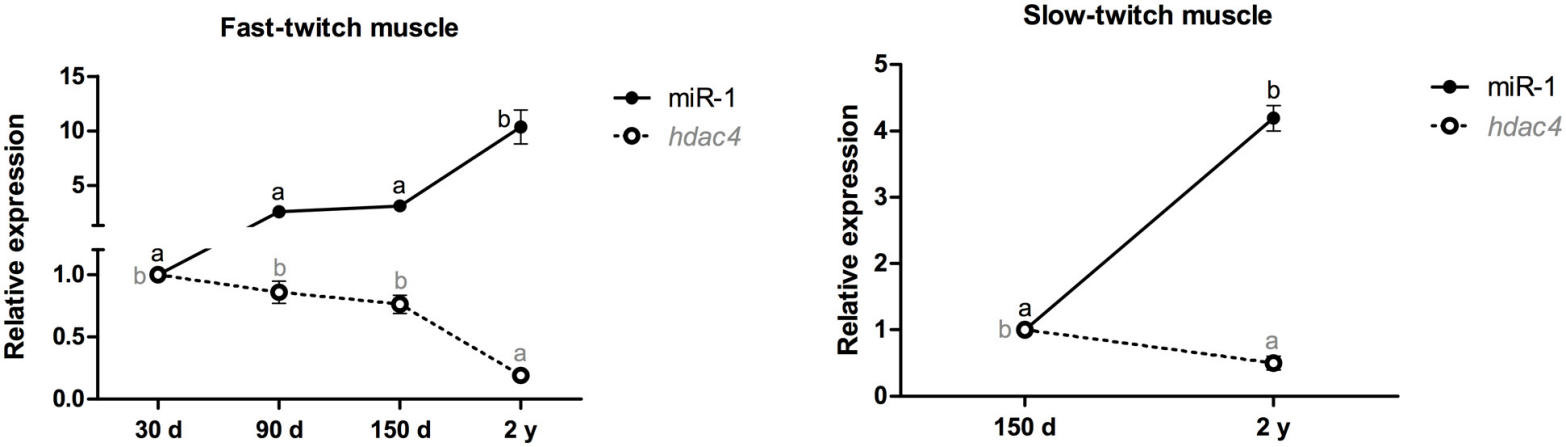
Relative expression of miR-1 and the *hdac4* mRNA in fast- and slow-twitch muscles of pacu during growth. miR-1 and *hdac4* mRNA expression was assessed by qPCR in pacu skeletal muscle at different development stages: 30-day-old larvae (30 d), 90-day-old juveniles (90 d), 150-day-old juveniles (150 d) and 2-year-old adults (2 y). The slow-twitch muscle was analyzed only in the 150-day-old and 2-year-old pacus, given the difficulty in isolating this tissue in younger animals (30- and 90-day-old pacus). The data are expressed as the fold change compared with the expression level in the fast-twitch muscle in the 30-day-old pacus or the expression level in the slow-twitch muscle in the 150-day-old pacus. The data are presented as the mean ± SEM (n = 6). The different letters indicate significant differences in expression (p<0.05).

The expression of miR-1 in the fast-twitch muscle was upregulated 10-fold in 2-year-old pacus (p<0.001), unlike the expression of the *hdac4* mRNA, which showed a 5-fold decrease in 2-year-old pacus (p<0.001). This variation was also observed in the slow-twitch muscle, wherein miR-1 was upregulated 4-fold in 2-year-old pacus (p<0.001), and the *hdac4* mRNA was downregulated 2-fold in these animals (p<0.05) (Fig 1).

**Fig 2:**
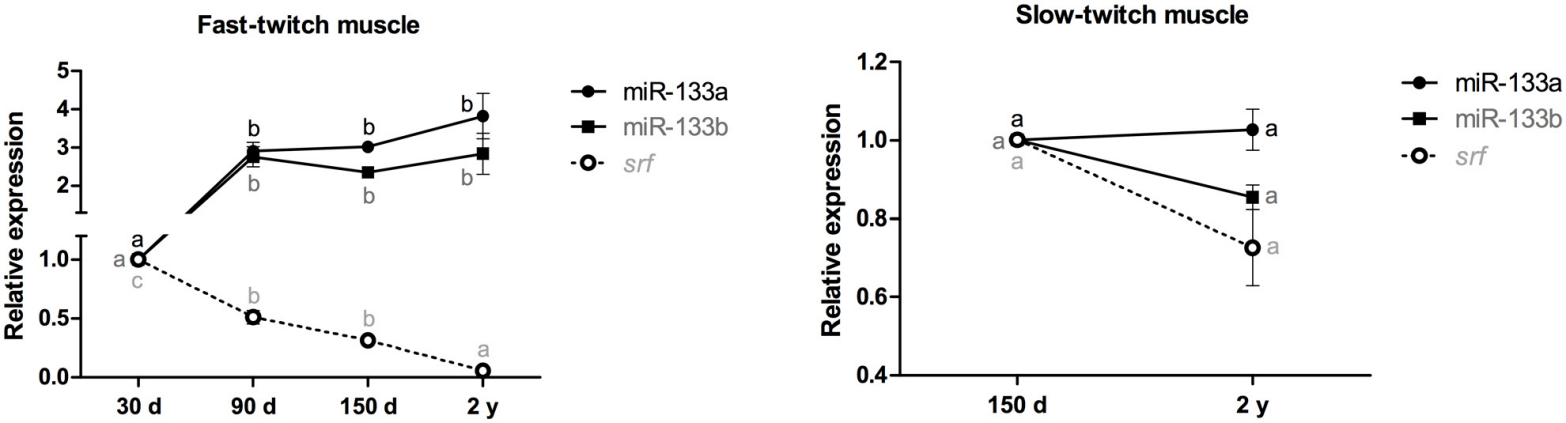
Relative expression of miR-133a, miR-133b and the *srf* mRNA in fast- and slow-twitch muscles of pacu during growth. miR-133a, miR-133b and *srf* mRNA expression was assessed by qPCR in pacu skeletal muscle at different development stages: 30-day-old larvae (30 d), 90-day-old juveniles (90 d), 150-day-old juveniles (150 d) and 2-year-old adults (2 y). The slow-twitch muscle was analyzed only in the 150-day-old and 2-year-old pacus, given the difficulty in isolating this tissue in younger animals (30- and 90-day-old pacus). The data are expressed as the fold change compared with the expression level in the fast-twitch muscle in the 30-day-old pacus or the expression level in the slow-twitch muscle in the 150-day-old pacus. The data are presented as the mean ± SEM (n = 6). The different letters indicate significant differences in expression (p<0.05).

In fast-twitch muscle, miR-133a and miR-133b exhibited a 2- to 4-fold increase in the 90-day-old, 150-day-old and 2-year-old animals (p<0.05), whereas the *srf* mRNA exhibited a 2- to 3-fold decrease in the 90- and 150-day-old animals (p<0.001) and a 17-fold decrease in the 2-year-old pacus (p<0.001). The differences in the expression of miR-133a, miR-133b and the *srf* mRNA in slow-twitch muscle between the 150-day-old and 2-year-old pacus were not significant (p>0.05) (Fig 2).

**Fig 3:**
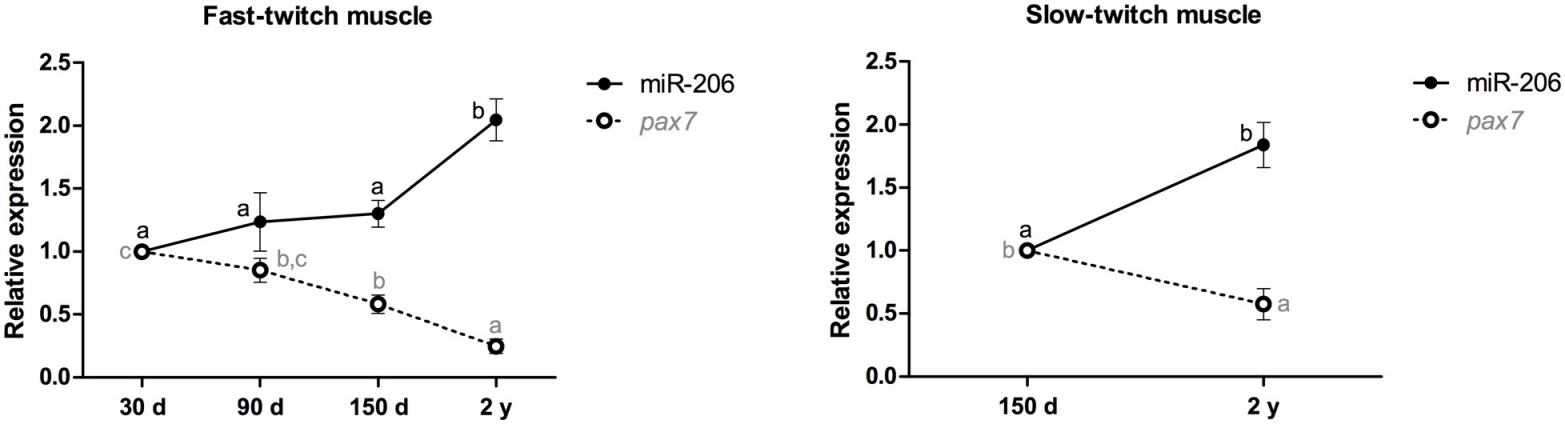
Relative expression of miR-206 and the *pax7* mRNA in fast- and slow-twitch muscles of pacu during growth. miR-206 and *pax7* mRNA expression was assessed by qPCR in pacu skeletal muscle at different development stages: 30-day-old larvae (30 d), 90-day-old juveniles (90 d), 150-day-old juveniles (150 d) and 2-year-old adults (2 y). The slow-twitch muscle was analyzed only in the 150-day-old and 2-year-old pacus, given the difficulty in isolating this tissue in younger animals (30- and 90-day-old pacus). The data are expressed as the fold change compared with the expression level in the fast-twitch muscle in the 30-day-old pacus or the expression level in the slow-twitch muscle in the 150-day-old pacus. The data are presented as the mean ± SEM (n = 6). The different letters indicate significant differences in expression (p<0.05).

miR-206 expression in fast-twitch muscle was upregulated 2-fold in 2-year-old pacus (p<0.01). By contrast, *pax7* mRNA expression was downregulated almost 2-fold in 150-day-old pacus (p<0.01) and 4-fold in 2-year-old animals (p<0.001). The same difference was observed in slow-twitch muscle, with a 2-fold increase in miR-206 in 2-year-old pacus (p<0.01) and an almost 2-fold decrease in the *pax7* mRNA in these fish (p<0.05) (Fig 3).

**Fig 4:**
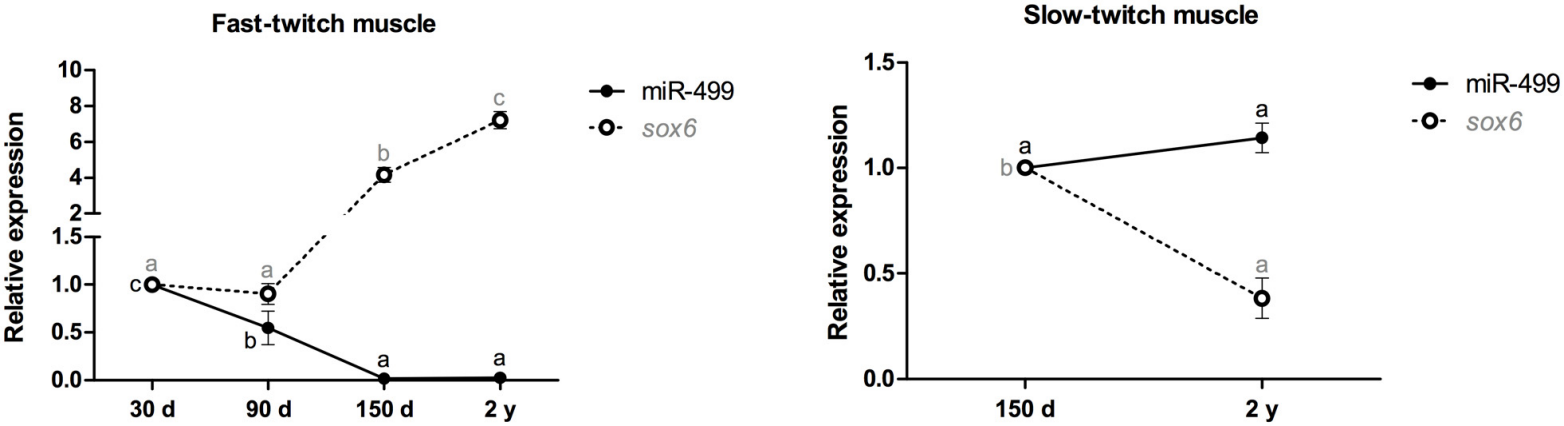
Relative expression of miR-499 and the *sox6* mRNA in fast- and slow-twitch muscles of pacu during growth. miR-499 and *sox6* mRNA expression was assessed by qPCR in pacu skeletal muscle at different development stages: 30-day-old larvae (30 d), 90-day-old juveniles (90 d), 150-day-old juveniles (150 d) and 2-year-old adults (2 y). The slow-twitch muscle was analyzed only in the 150-day-old and 2-year-old pacus, given the difficulty in isolating this tissue in younger animals (30- and 90-day-old pacus). The data are expressed as the fold change compared with the expression level in the fast-twitch muscle in the 30-day-old pacus or the expression level in the slow-twitch muscle in the 150-day-old pacus. The data are presented as the mean ± SEM (n = 6). The different letters indicate significant differences in expression (p<0.05).

In fast-twitch muscle, the expression of miR-499 was decreased by 33- to 50-fold in 150-day-old and 2-year-old pacus (p<0.001), and there was a 2-fold decrease in 90-day-old animals (p<0.05). The *sox6* mRNA was upregulated 4-fold at 150 days (p<0.001) and 7-fold at 2 years (p<0.001). In slow-twitch muscle, the miR-499 levels in the 150-day-old and 2-year-old pacus were not significantly different (p = 0.37), whereas the expression of the *sox6* mRNA was downregulated 2.5-fold in 2-year-old pacus (p<0.001) (Fig 4).

**Fig 5:**
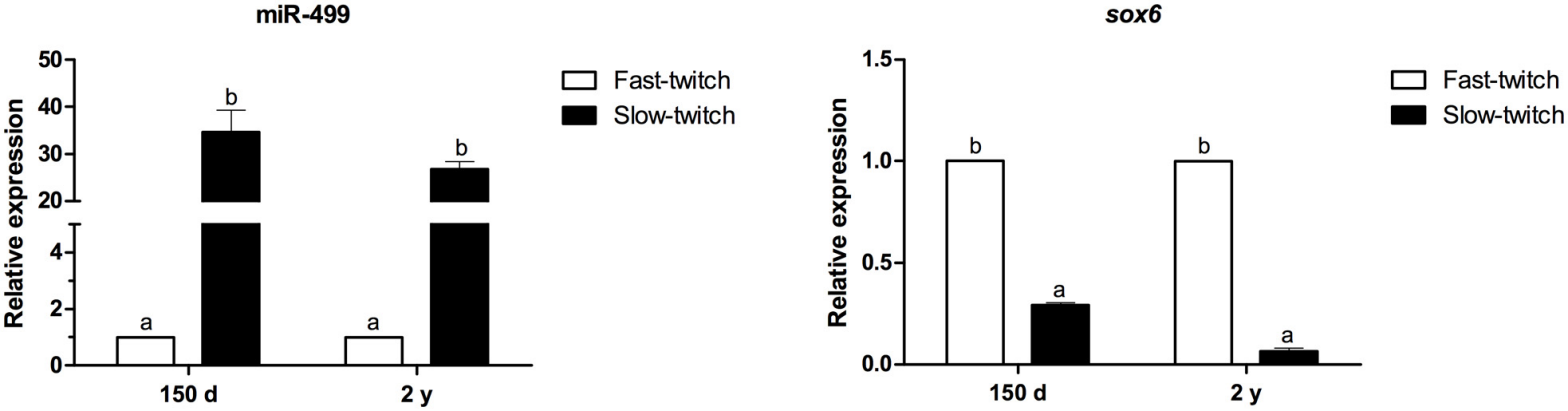
Relative expression of miR-499 and the *sox6* mRNA between fast- and slow-twitch muscles in 150-day-old and 2-year-old pacus. miR-499 and *sox6* mRNA expression was assessed by qPCR in skeletal muscle from 150-day-old juvenile (150 d) and 2-year-old adult (2 y) pacus. The skeletal muscle was analyzed only in the 150-day-old and 2-year-old pacus given the difficulty in isolating the slow-twitch muscle in younger animals (30- and 90-day-old pacus). The data are expressed as the fold change compared with the expression level in the fast-twitch muscle. The data are presented as the mean ± SEM (n = 6). The different letters indicate significant differences in expression (p<0.05).

Given the involvement of miR-499 in the specification and maintenance of muscle fiber phenotypes, the expression of this miRNA and the *sox6* mRNA was also compared between the pacu fast- and slow-twitch muscles. The expression of miR-499 was markedly higher in slow-twitch muscle, both at 150 days (35-fold increase; p<0.001) and at 2 years (27-fold increase; p<0.001). Moreover, the *sox6* mRNA levels were significantly lower in slow-twitch muscle, both at 150 days (3-fold decrease; p<0.001) and at 2 years (17-fold decrease; p<0.001) (Fig 5).

To complement our study, we performed a comparison of the miRNA target sites at the 3’UTR of the examined mRNAs between different species of vertebrates (*Homo sapiens*, *Mus musculus*, *Danio rerio* and *Piaractus mesopotamicus*) using the *MAFFT version 7 software* (http://mafft.cbrc.jp/alignment/server/index.html) [25] and *miRmap software* (http://mirmap.ezlab.org/app/) [26] (S3 File). We also analyzed the miRNA seed sequences between several species using the *Geneious software 4*.*8*.*5* (http://www.geneious.com/) [27] (S4 File). These comparisons revealed a high conservation of the nucleotide sequences, which contributes to confirm the interaction between the miRNAs and their respective targets in skeletal muscle of pacu and their likely regulatory roles in muscle growth and in the maintenance of the muscle phenotype. Moreover, we performed a bioinformatic prediction of the interactions between miR-1/*hdac4*, miR-133a/*srf*, miR-133b/*srf*, miR-206/*pax7* and miR-499/*sox6* using the *RNAhybrid software* (http://bibiserv.techfak.uni-bielefeld.de/rnahybrid/) [28], which is a tool for finding the minimum free energy (MFE) of hybridization and provides potential binding sites through nucleotide base complementarity. The MFEs were within accepted ranges (S5 File).

### Myoblast cell culture

**Fig 6:**
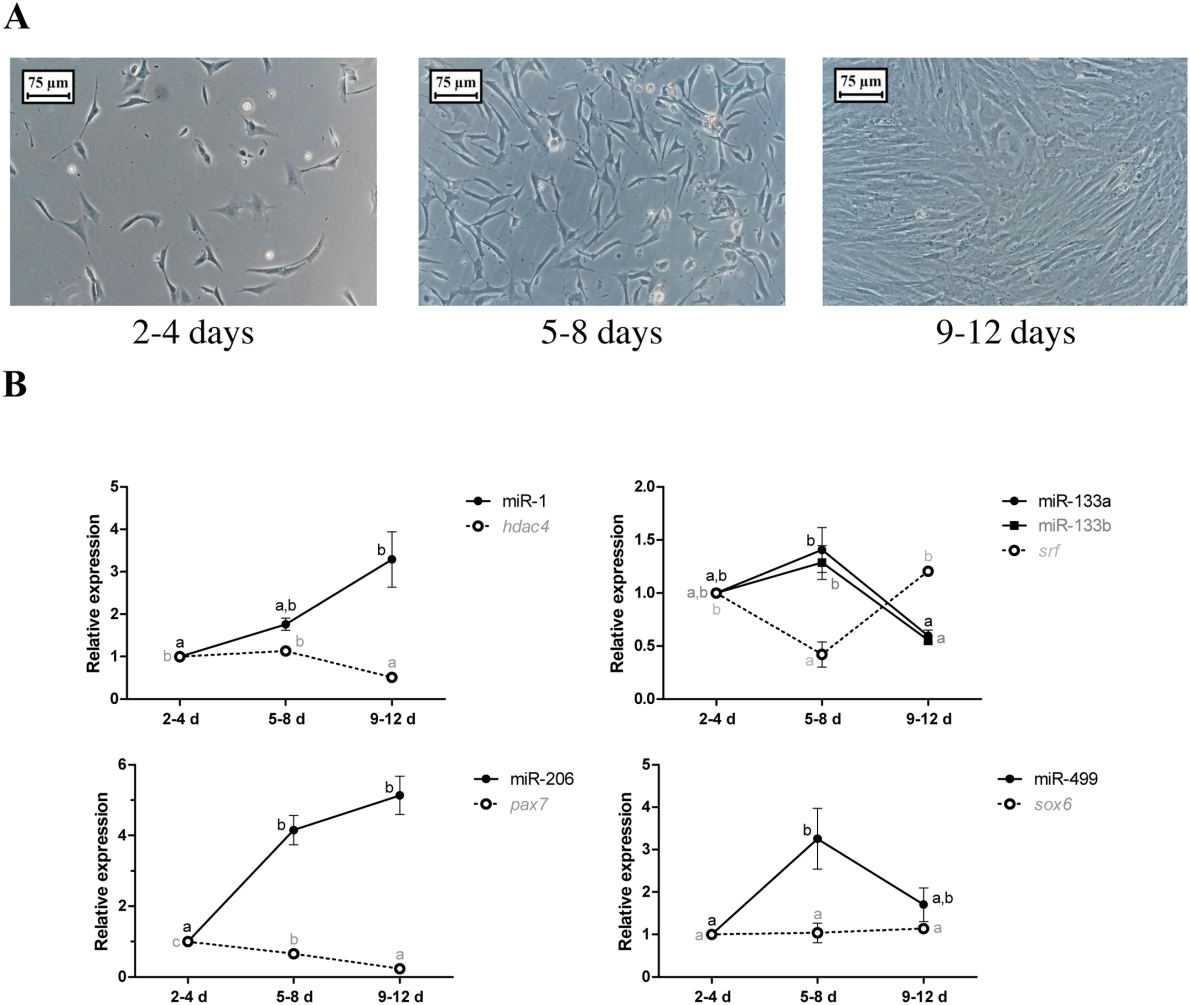
Relative expression of the miRNAs and their respective targets in pacu myoblast cell cultures. (A) The primary myoblast cultures isolated from the fast-twitch muscle of juvenile pacus were studied at different phases: 2–4 days (2–4 d), 5–8 days (5–8 d) and 9–12 days (9–12 d). The images were obtained on an inverted microscope with 20x magnification (Bars: 75 μm). (B) The miRNAs and target mRNAs expression was assessed by qPCR. The data are expressed as the fold change compared with the expression level at 2–4 days. The data are presented as the mean ± SEM from 3 independent cell cultures. The different letters indicate significant differences in expression (p<0.05).

To provide further insight and to complement our study, we performed a pacu myoblast cell culture, which allowed us to investigate miRNAs in a controlled environment. We evaluated the expression of the miRNAs and their targets in a progression from myoblast proliferation to differentiation by studying the cell cultures at 2–4 days (commitment and early proliferation), 5–8 days (late proliferation and early fusion) and 9–12 days (late fusion and myotube formation) (Fig 6A). The expression of miR-1 was upregulated 3-fold at 9–12 days (p<0.05), whereas the expression of the *hdac4* mRNA was the opposite, with a 2-fold decrease at 9–12 days (p<0.05). The expression of miR-133a and miR-133b were quite the same, with a significant increase at 5–8 days compared with that at 9–12 days (1.7-fold decrease from 2–4 days; p<0.05). By contrast, the expression of the *srf* mRNA was downregulated 2.5-fold at 5–8 days (p<0.05). miR-206 exhibited a 4- to 5-fold increase at 5–8 days and 9–12 days (p<0.01), whereas the expression of the *pax7* mRNA was downregulated 1.5-fold at 5–8 days and 5-fold at 9–12 days (p<0.05). The expression of miR-499 was upregulated 3-fold at 5–8 days (p<0.05), and the *sox6* mRNA levels in the different cell culture phases were not significantly different (p = 0.88) (Fig 6B).

### MRF expression

**Fig 7:**
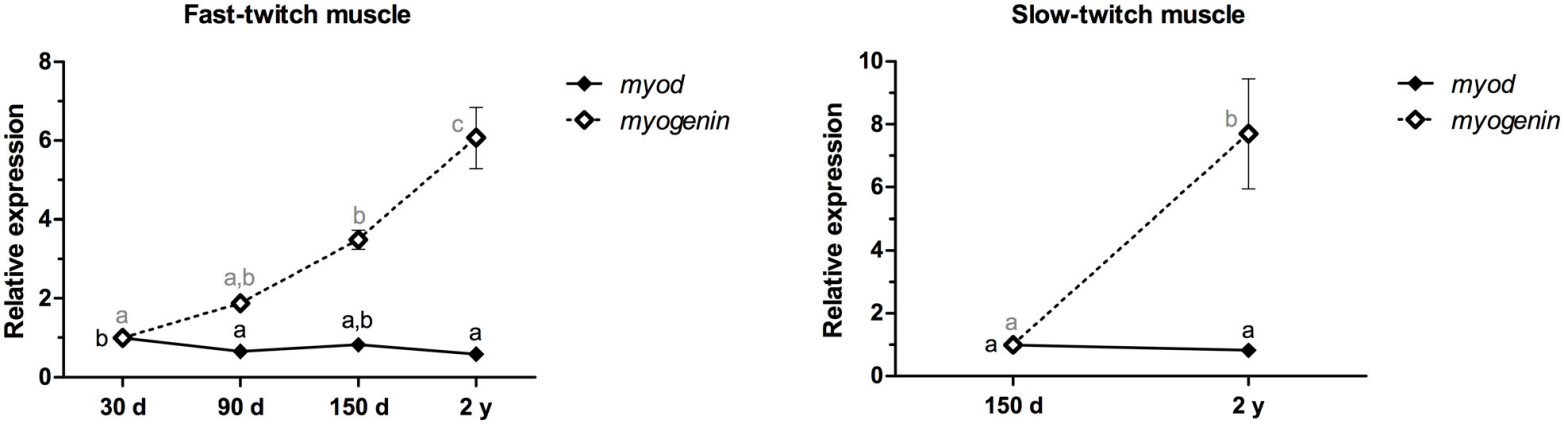
Relative expression of the *myod* and *myogenin* mRNAs in fast- and slow-twitch muscles of pacu during growth. *myod* and *myogenin* mRNAs expression was assessed by qPCR in pacu skeletal muscle at different development stages: 30-day-old larvae (30 d), 90-day-old juveniles (90 d), 150-day-old juveniles (150 d) and 2-year-old adults (2 y). The slow-twitch muscle was analyzed only in the 150-day-old and 2-year-old pacus, given the difficulty in isolating this tissue in younger animals (30- and 90-day-old pacus). The data are expressed as the fold change compared with the expression level in the fast-twitch muscle in the 30-day-old pacus or the expression level in the slow-twitch muscle in the 150-day-old pacus. The data are presented as the mean ± SEM (n = 6). The different letters indicate significant differences in expression (p<0.05).

Skeletal muscle growth is regulated by the sequential expression of MRFs, including MyoD and Myogenin, which are involved in myoblast proliferation and differentiation, respectively [7]. We also analyzed the expression of the *myod* and *myogenin* mRNAs during the growth of fast- and slow-twitch muscles and in pacu myoblast cell cultures to confirm that the proliferation and differentiation mechanisms were present. The expression of the *myod* mRNA in fast-twitch muscle was downregulated 1.5- and 1.7-fold in the 90-day-old and 2-year-old pacus (p<0.01), respectively. The *myogenin* mRNA expression was increased 3.5-fold in 150-day-old animals (p<0.01) and 6-fold in 2-year-old pacus (p<0.001). In slow-twitch muscle, the expression of the *myod* mRNA in 150-day-old and 2-year-old pacus was not significantly different (p = 0.34), whereas the expression of the *myogenin* mRNA was upregulated almost 8-fold in 2-year-old pacus (p<0.05) (Fig 7).

**Fig 8:**
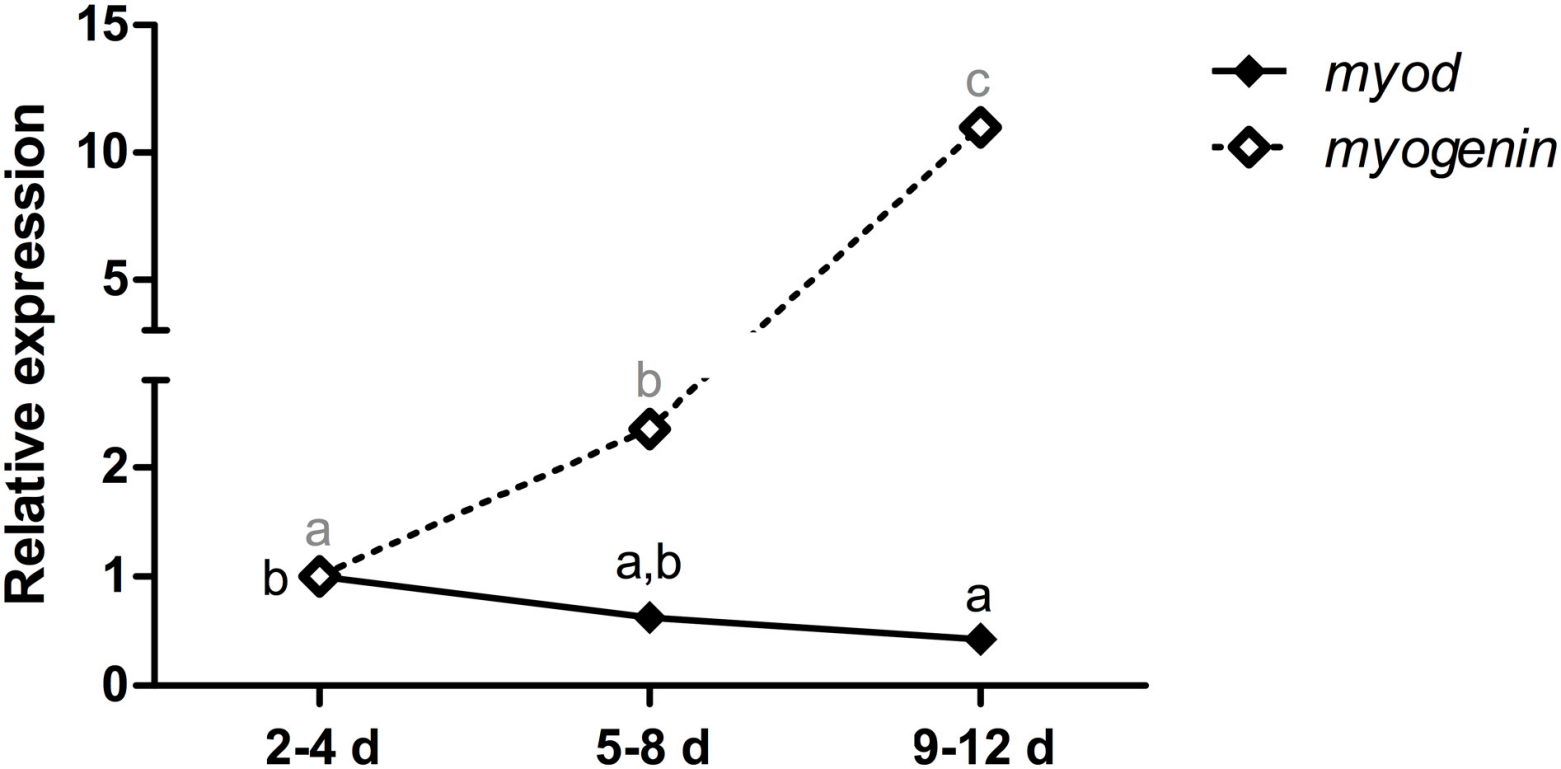
Relative expression of the *myod* and *myogenin* mRNAs in pacu myoblast cell cultures. *myod* and *myogenin* mRNAs expression was assessed by qPCR in primary myoblast cultures isolated from fast-twitch muscle of juvenile pacus at different phases: 2–4 days (2–4 d), 5–8 days (5–8 d) and 9–12 days (9–12 d). The data are expressed as the fold change compared with the expression level at 2–4 days. The data are presented as the mean ± SEM from 3 independent cell cultures. The different letters indicate significant differences in expression (p<0.05).

The expression of the *myod* mRNA in myoblast cell cultures was downregulated 2.5-fold at 9–12 days (p<0.05), whereas the expression of the *myogenin* mRNA was increased 2-fold at 5–8 days (p<0.05) and 11-fold at 9–12 days (p<0.001) (Fig 8).

## Discussion

### miRNAs and target expression

A more general analysis of the expression results allows us to affirm that the miR-1, miR-133a, miR-133b and miR-206 levels increased with the growth of the pacus, whereas the *hdac4*, *srf* and *pax7* mRNA levels decreased with the progression of muscle growth. By contrast, miR-499 and the *sox6* mRNA exhibited a decrease and an increase in their expression levels, respectively, during fast-twitch muscle growth, whereas the *sox6* mRNA was decreased in slow-twitch muscle during growth. Thus, it is possible to observe an inverse correlation between the expression of the miRNAs and the expression of their target mRNAs. This makes sense, given the biological function of the miRNAs within the cells, and suggests that miR-1, miR-133a, miR-133b, miR-206 and miR-499 act directly on the *hdac4*, *srf*, *pax7* and *sox6* mRNAs in pacu skeletal muscle. Similar results were observed in the fish species *Oreochromis niloticus* [18,20], and an increased expression of these miRNAs was also observed during *Cyprinus carpio* growth [29].

Hdac4, which is a member of the class II histone deacetylases, associates with the transcription factor Mef2 (*myocyte enhancer factor 2*) through its N-terminal tail. This binding represses the activity of Mef2, an essential factor for the terminal differentiation of myoblasts [30,31]. The lower levels of the *hdac4* mRNA in the fast- and slow-twitch muscles of adult pacus, most likely due to silencing by the increased expression of miR-1, promoted Mef2 action and therefore muscle differentiation. Moreover, the high expression of the *hdac4* mRNA in the fast- and slow-twitch muscles of younger pacus inhibited the regulation of Mef2-dependent genes, contributing to the maintenance of myoblasts in a proliferative state in these animals.

Srf, which belongs to the MADS box transcription factor family, is a central regulator of the cell cycle and promotes the transcriptional activation of immediate-early genes by binding to serum response elements (Sre) in their promoters [32]. However, in addition to this role in the proliferation of several cell types, Srf also regulates the expression of muscle-specific genes, such as *α-actin*, *dystrophin* and *myosin light chain*, by binding to a nucleotide sequence known as the CArG box, which demonstrates its activity in muscle differentiation as well [33]. In fast-twitch muscle, we presume that Srf activated the immediate-early genes in larval pacus, promoting myoblast proliferation. Beginning at 90 days, Srf began to stimulate muscle differentiation, but the high levels of miR-133a and miR-133b silenced its expression to maintain cellular proliferation. In adult pacus, we observed increased expression of miR-133a and miR-133b and low levels of the *srf* mRNA, which was surprising because myoblast differentiation is accompanied by muscle maturation, a process that gradually increases with muscle growth. It is likely that the systemic variations that exist in the *in vivo* approach did not clarify the stimulating role of these miRNAs and the *srf* mRNA in myoblast proliferation and differentiation, respectively. This uncertainty emphasizes the importance of myoblast cell culture studies [6] to evaluate the expression of regulatory molecules in a controlled environment. We did not observe significant differences in the expression of miR-133a, miR-133b and the *srf* mRNA during slow-twitch muscle growth, but this result is likely because we did not have this tissue from the 30- and 90-day-old pacus. We presume that, similar to fast-twitch muscle, the main function of the *srf* mRNA in the slow-twitch muscle of the 150-day-old and 2-year-old pacus was to stimulate myoblast differentiation, but a basal level of myoblast proliferation was maintained by miR-133a and miR-133b.

Pax7 is a transcription factor that is expressed in satellite cells [34], which promote the postnatal growth of skeletal muscle. This factor is required for the survival of satellite cells and for the subsequent proliferation of myoblasts, in addition to preventing precocious differentiation [35,36]. The expression of the *pax7* mRNA was higher in the fast-twitch muscle of 30- and 90-day-old pacus and subsequently decreased, likely as a result of the rising levels of miR-206, which contributed to the increasingly prominent differentiation. This pattern was also observed in slow-twitch muscle, with increasing levels of miR-206 and decreasing levels of the *pax7* mRNA, promoting increased myoblast differentiation in adult pacus.

miR-499 participates in the specification and maintenance of twitch phenotypes. van Rooij et al. (2009) [13] demonstrated that the increased expression of miR-499 in the skeletal muscle of mice was sufficient to promote the transition of fast-twitch to slow-twitch muscle fibers, which was mediated in part by the downregulation of *sox6*. In zebrafish [19] and Nile tilapia [20], miR-499 mediates the translational repression of Sox6, which represses slow-twitch-specific genes, such as *slow myosin heavy chain 1* and *slow troponin* [37], in a manner that is involved in the maintenance of the fast-twitch phenotype in muscle fibers. We observed higher miR-499 expression and low *sox6* mRNA expression in the fast-twitch muscle of larval pacus, likely because the fibers were in the process of forming. As the fibers matured and differentiated, particularly in the adult pacus, the expression of the *sox6* mRNA also increased, likely as a result of the decreased levels of miR-499, allowing the full definition of the fast-twitch phenotype. In slow-twitch muscle, there was no significant difference in the miR-499 levels between 150-day-old and 2-year-old pacus, but its levels likely decreased the expression of the *sox6* mRNA in adult pacus. This low level of the *sox6* mRNA demonstrates the increasing specification of the slow-twitch phenotype. These results were corroborated by the expression of miR-499 and the *sox6* mRNA between fast- and slow-twitch muscles of the 150-day-old and 2-year-old pacus. The higher expression of miR-499 in slow-twitch muscle at both development stages may have reduced the levels of the *sox6* mRNA and contributed to the establishment of the slow-twitch phenotype in the muscle fibers. By contrast, the *sox6* mRNA was expressed at higher levels in the fast-twitch muscle of the 150-day-old and 2-year-old pacus, contributing to the definition of the fast-twitch phenotype.

In summary, the high levels of miR-1 and miR-206 in adult pacus may have promoted myoblast differentiation in the fast- and slow-twitch muscles of these animals through the silencing of the *hdac4* and *pax7* mRNAs, respectively. On the other hand, the expression of miR-133a and miR-133b increased earlier, beginning at 90 days, which may have silenced the *srf* mRNA to maintain the proliferation of myoblasts in juvenile pacus. miR-499 may have regulated the establishment of the slow-twitch phenotype in muscle fibers through the silencing of the *sox6* mRNA.

The pacu myoblast cell cultures also revealed an inverse correlation between the expression of the miRNAs and their targets, similar to that observed in fast- and slow-twitch muscles during growth. miR-1 and miR-206 expression was increased during myoblast differentiation (9–12 days), which reduced the levels of the *hdac4* and *pax7* mRNAs in this phase of cell culture. Given the roles of the *hdac4* and *pax7* mRNAs in repressing myoblast differentiation, their increased expression during early (2–4 days) and late proliferation (5–8 days) makes sense. The expression of miR-133a and miR-133b was higher during myoblast proliferation (2–4 and 5–8 days), whereas the *srf* mRNA was expressed at lower levels at 5–8 days. The *srf* mRNA started to stimulate the mechanism of proliferation at 2–4 days, but its level decreased at 5–8 days due to the high expression of miR-133a and miR-133b. Its expression rose again at 9–12 days, when *srf* promoted myoblast differentiation. These results contrast with the data from the fast-twitch muscles during growth. However, the regulation of myoblast proliferation by miR-133a and miR-133b and the role of the *srf* mRNA in myoblast differentiation at the end of growth became more evident in myoblast cell cultures, showing that this technique is very useful to expand our knowledge of the molecular mechanisms that regulate muscle growth [6]. The expression levels of miR-499 were higher at 5–8 days, and the *sox6* mRNA was not expressed at significantly different levels during myoblast proliferation and differentiation. The innervation pattern is essential for the specification of twitch phenotypes in muscle fibers; a tonic and low-frequency neural stimulation induces the slow-twitch phenotype, whereas a phasic and high-frequency neural stimulation promotes the fast-twitch phenotype [38]. However, the lack of innervation in primary myoblast cell cultures makes the specification and maintenance of twitch phenotypes markedly different from what occurs *in vivo*. It is possible that miR-499 and the *sox6* mRNA did not participate in the definition of the twitch phenotypes during progression of myoblast cell cultures, but may have participated in another biological process. These experiments in pacu myoblast cell cultures strengthened our work and contributed to the elucidation of the roles of miRNAs in myoblast proliferation and differentiation, as well as also suggested the direct silencing of the *hdac4*, *srf* and *pax7* mRNAs by miR-1, miR-133a, miR-133b and miR-206, respectively, in pacu skeletal muscle.

### MRF expression

In fast-twitch muscle, the *myod* mRNA levels were slightly decreased throughout pacu growth, whereas the *myogenin* mRNA levels were increased during growth. The increased expression of the *myod* mRNA in larval pacus promoted intense myoblast proliferation, which is required for the large-scale formation of muscle fibers that occurs in this development stage. In juvenile pacus, hyperplasia and hypertrophy simultaneously occurred [39], allowing for the formation of some fibers, whereas others increased in size. These processes were regulated by both the *myod* mRNA, which was decreased at 90 days, and the *myogenin* mRNA, which was increased at 150 days. The *myogenin* mRNA was expressed at higher levels in adult pacus and promoted marked differentiation at this development stage [40]. This result makes sense because the fibers become increasingly mature and muscle growth rates begin to decline as the fish approach their final size. In slow-twitch muscle, the expression levels of the *myod* mRNA in the 150-day-old and 2-year-old pacus were not different, and the *myogenin* mRNA was also expressed at higher levels in adult pacus, which corroborates the data obtained in the fast-twitch muscle. The experiments in myoblast cell cultures confirmed the roles of the *myod* and *myogenin* mRNAs in proliferation and differentiation, respectively, because the expression of the *myod* mRNA was decreased with the progression of the cell cultures, with higher levels at 2–4 and 5–8 days, whereas the *myogenin* mRNA levels increased, with higher expression at 9–12 days.

Our results suggest that during muscle growth, the *myod* mRNA regulated myoblast proliferation, which appeared more pronounced in the early development stages and tended to decrease with growth progression. In late development stages, the *myogenin*-mediated myoblast differentiation became more predominant.

## Conclusions

**Fig 9:**
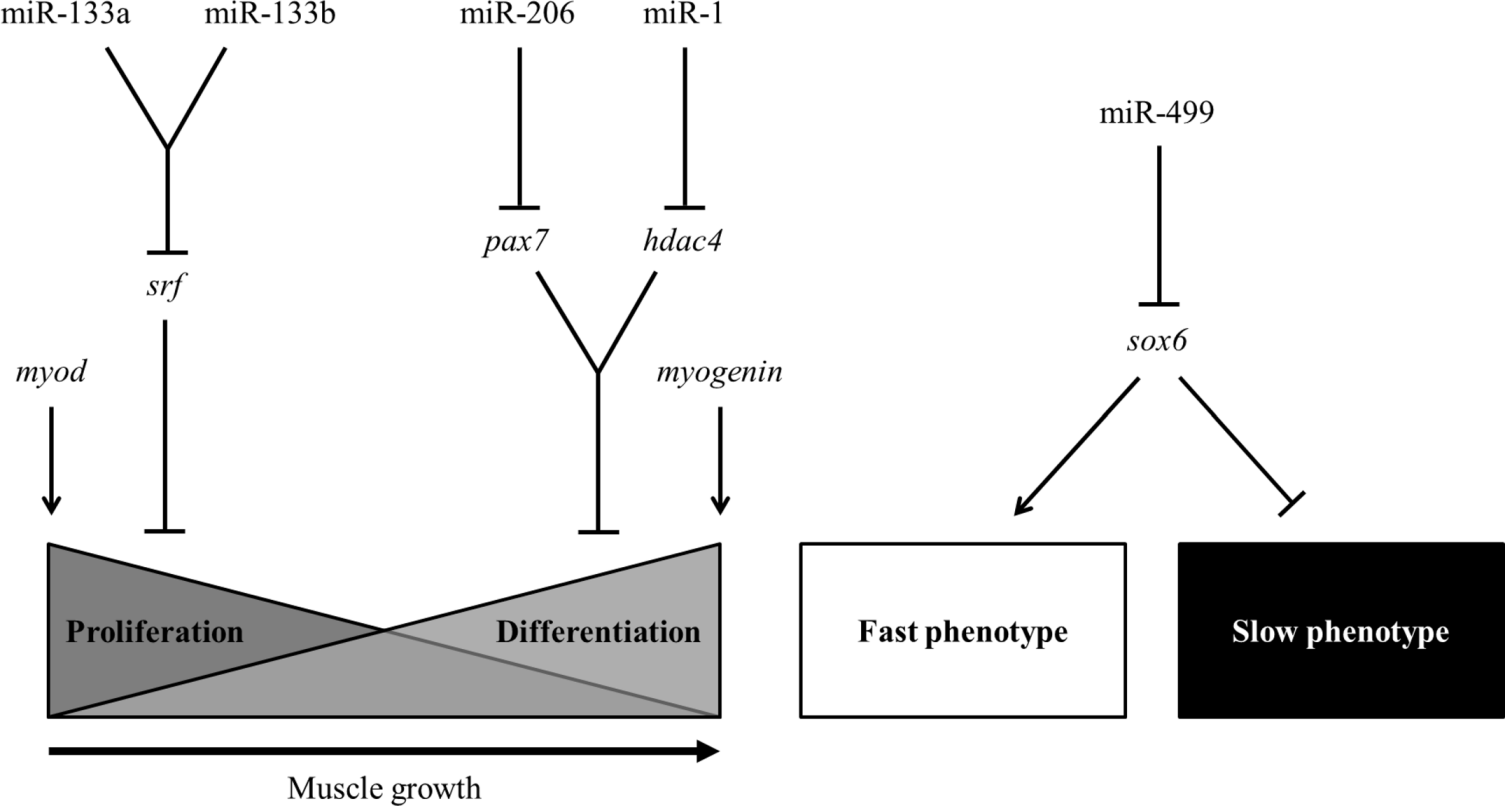
Model of MRF- and miRNA-mediated regulation of pacu skeletal muscle. Skeletal muscle growth of pacu is regulated by miR-1, miR-133a, miR-133b and miR-206, which likely modulate myoblast proliferation and differentiation by silencing of the *hdac4*, *srf* and *pax7* mRNAs. The specification and maintenance of the twitch phenotypes are likely regulated by miR-499 through the silencing of the *sox6* mRNA.

The expression patterns of miR-1, miR-133a, miR-133b, miR-206 and miR-499 indicate a possible role in the regulation of skeletal muscle in pacu, controlling factors involved in the myoblast proliferation and differentiation, and in the specification and maintenance of the twitch phenotypes in muscle fibers (Fig 9).

Even with the important findings of our study, it is possible that miR-1, miR-133, miR-206 and miR-499 are also involved in other aspects of muscle development, such as skeletal muscle performance and metabolism [41,42], because there are dozens of target mRNAs that are regulated by a single miRNA. Further investigations are necessary to increase our understanding of the roles of each miRNA, particularly through gain and loss of function assays that can be performed in primary myoblast cell cultures [6]. The expression of these miRNAs exhibited variations between the different development stages and between distinct twitch phenotypes, and this study provides the first expression profile of the miRNAs in the skeletal muscle of pacu. These results could serve as the basis for further research on fish muscle growth and may assist in the development of new farming strategies that induce increased muscle mass and ensure greater production.

## Supporting Information

S1 File Additional information about the qPCR data for the miRNAs, their target mRNAs and the reference genes. (PDF)

S2 File Extent of the variations in the expression of the reference genes by qPCR. The Ct (threshold cycle) values of the *U6 snRNA* and *18S rRNA* were similar between all groups.
(PDF)

S3 File miRNA binding sites at the 3’UTR of the target mRNAs between different species of vertebrates. The seed-matched sequences at the 3’UTR of the mRNAs are shown in red, the conserved regions between the aligned sequences are indicated by stars, and the dashes represent the manually inserted gaps.
(PDF)

S4 File Seed regions in the mature sequences of the miRNAs between several species. The seed sequences of the miRNAs are in the purple box, and the fish species are in the gray box.
(PDF)

S5 File Bioinformatic prediction of the interactions between the miRNAs and their target mRNAs. The MFE (minimum free energy) values were within accepted ranges.
(PDF)

S1 Table *TaqMan* ^*®*^ assays used for the miRNA and *U6 snRNA* amplification by qPCR. (PDF)

S2 Table Primers used for *hdac4*, *srf*, *pax7*, *sox6*, *myod*, *myogenin* and *18S* mRNA amplification by qPCR. (PDF)
