# Correction: Differential microRNA Expression in Fast- and Slow-Twitch Skeletal Muscle of *Piaractus mesopotamicus* during Growth

**DOI:** 10.1371/journal.pone.0144481

**Published:** 2015-12-02

**Authors:** The PLOS ONE Staff

The ninth author’s name is spelled incorrectly. The correct name is: Maeli Dal-Pai-Silva. The publisher apologizes for the error.
