# Supplementary material for: Differential microRNA Expression in Fast- and Slow-Twitch Skeletal Muscle of Piaractus mesopotamicus during Growth
Source: PLoS One. 2015 Nov 3;10(11):e0141967. doi: 10.1371/journal.pone.0141967 (PMC4631509; doi:10.1371/journal.pone.0141967)
Supplement: S1 File — (PDF) [file pone.0141967.s001.pdf]

miR-1 (fast-twitch muscle)							
**Group**	**Sample**	**Ct mean**	**SD of 6 replicates**	**Average fold change**	**SEM**	**95% CI (Lower-Upper)**	**F value**
30 days	1	19,28	0,34	1	0,09	0,76-1,24	26,46
	2	18,77					
	3	18,97					
	4	19,17					
	5	18,43					
	6	18,57					
90 days	7	17,02	0,47	2,57	0,34	1,70-3,44	
	8	17,64					
	9	17,23					
	10	18,34					
	11	17,80					
	12	17,88					
150 days	13	17,03	0,23	3,11	0,19	2,61-3,60	
	14	17,53					
	15	17,69					
	16	17,40					
	17	17,24					
	18	17,33					
2 years	19	14,84	0,56	10,35	1,57	6,32-14,37	
	20	15,63					
	21	16,13					
	22	16,07					
	23	16,19					
	24	15,19					

Ct mean: threshold cycle mean of 2 replicates;
SD: standard deviation;
Average fold change: average change in expression relative to 30 days group;
SEM: standard error of mean;
CI: confidence interval.

hdac4 (fast-twitch muscle)							
**Group**	**Sample**	**Ct mean**	**SD of 6 replicates**	**Average fold change**	**SEM**	**95% CI (Lower-Upper)**	**F value**
30 days	1	17,39	0,20	1	0,08	0,79-1,21	24,48
	2	17,57					
	3	17,47					
	4	17,04					
	5	17,60					
	6	17,35					
90 days	7	17,79	0,13	0,86	0,09	0,63-1,09	
	8	17,64					
	9	17,46					
	10	17,68					
	11	17,46					
	12	17,52					
150 days	13	17,68	0,20	0,76	0,07	0,58-0,95	
	14	17,94					
	15	17,45					
	16	17,85					
	17	17,68					
	18	18,00					
2 years	19	19,72	0,31	0,19	0,03	0,13-0,26	
	20	19,59					
	21	19,55					
	22	19,84					
	23	20,20					
	24	20,28					

Ct mean: threshold cycle mean of 2 replicates;

SD: standard deviation;

Average fold change: average change in expression relative to 30 days group;

SEM: standard error of mean;

CI: confidence interval.

miR-133a (fast-twitch muscle)							
**Group**	**Sample**	**Ct mean**	**SD of 6 replicates**	**Average fold change**	**SEM**	**95% CI (Lower-Upper)**	**F value**
30 days	1	18,23	0,18	1	0,04	0,89-1,11	13,49
	2	17,76					
	3	18,07					
	4	17,95					
	5	17,76					
	6	17,89					
90 days	7	16,16	0,28	2,90	0,22	2,33-3,48	
	8	16,65					
	9	16,33					
	10	16,99					
	11	16,60					
	12	16,51					
150 days	13	16,16	0,24	3,02	0,15	2,63-3,41	
	14	16,35					
	15	16,55					
	16	16,50					
	17	16,65					
	18	16,85					
2 years	19	15,42	0,59	3,82	0,59	2,30-5,34	
	20	16,04					
	21	16,61					
	22	16,75					
	23	16,83					
	24	15,71					

Ct mean: threshold cycle mean of 2 replicates;
SD: standard deviation;
Average fold change: average change in expression relative to 30 days group;
SEM: standard error of mean;
CI: confidence interval.

miR-133b (fast-twitch muscle)							
**Group**	**Sample**	**Ct mean**	**SD of 6 replicates**	**Average fold change**	**SEM**	**95% CI (Lower-Upper)**	**F value**
30 days	1	17,56	0,12	1	0,04	0,90-1,10	7,75
	2	17,26					
	3	17,48					
	4	17,48					
	5	17,34					
	6	17,33					
90 days	7	15,56	0,31	2,76	0,26	2,08-3,44	
	8	16,25					
	9	16,01					
	10	16,43					
	11	16,00					
	12	16,27					
150 days	13	16,08	0,26	2,35	0,12	2,03-2,67	
	14	16,09					
	15	16,27					
	16	16,40					
	17	16,44					
	18	16,77					
2 years	19	15,23	0,73	2,84	0,54	1,46-4,22	
	20	15,85					
	21	16,76					
	22	16,77					
	23	16,89					
	24	15,51					

Ct mean: threshold cycle mean of 2 replicates;

SD: standard deviation;

Average fold change: average change in expression relative to 30 days group;

SEM: standard error of mean;

CI: confidence interval.

***srf* (fast-twitch muscle)**							
**Group**	**Sample**	**Ct mean**	**SD of 6 replicates**	**Average fold change**	**SEM**	**95% CI (Lower-Upper)**	**F value**
30 days	1	16,85	0,35	1	0,09	0,77-1,23	47,70
	2	16,44					
	3	17,09					
	4	16,10					
	5	16,56					
	6	16,40					
90 days	7	17,17	0,61	0,51	0,06	0,37-0,66	
	8	18,24					
	9	17,37					
	10	18,31					
	11	17,16					
	12	16,85					
150 days	13	17,64	0,48	0,31	0,04	0,21-0,42	
	14	18,23					
	15	18,07					
	16	19,07					
	17	18,01					
	18	18,47					
2 years	19	20,27	0,76	0,06	0,01	0,02-0,09	
	20	20,19					
	21	20,37					
	22	21,93					
	23	21,50					
	24	21,46					

Ct mean: threshold cycle mean of 2 replicates;
SD: standard deviation;
Average fold change: average change in expression relative to 30 days group;
SEM: standard error of mean;
CI: confidence interval.

miR-206 (fast-twitch muscle)							
**Group**	**Sample**	**Ct mean**	**SD of 6 replicates**	**Average fold change**	**SEM**	**95% CI (Lower-Upper)**	**F value**
30 days	1	19,18	0,53	1	0,15	0,62-1,38	7,11
	2	18,76					
	3	18,03					
	4	18,03					
	5	17,91					
	6	18,80					
90 days	7	18,77	0,73	1,24	0,23	0,64-1,83	
	8	17,91					
	9	17,60					
	10	19,26					
	11	18,88					
	12	17,54					
150 days	13	17,71	0,34	1,30	0,11	1,03-1,57	
	14	18,22					
	15	17,89					
	16	18,23					
	17	18,36					
	18	18,65					
2 years	19	17,20	0,26	2,04	0,17	1,62-2,47	
	20	17,69					
	21	17,87					
	22	17,35					
	23	17,32					
	24	17,57					

Ct mean: threshold cycle mean of 2 replicates;
SD: standard deviation;
Average fold change: average change in expression relative to 30 days group;
SEM: standard error of mean;
CI: confidence interval.

***pax7* (fast-twitch muscle)**							
**Group**	**Sample**	**Ct mean**	**SD of 6 replicates**	**Average fold change**	**SEM**	**95% CI (Lower-Upper)**	**F value**
30 days	1	19,51	0,19	1	0,05	0,88-1,12	22,00
	2	19,12					
	3	19,51					
	4	19,10					
	5	19,45					
	6	19,36					
90 days	7	19,57	0,30	0,85	0,10	0,61-1,10	
	8	19,51					
	9	19,48					
	10	19,73					
	11	20,03					
	12	19,13					
150 days	13	20,50	0,44	0,58	0,07	0,39-0,77	
	14	20,64					
	15	19,85					
	16	20,05					
	17	20,33					
	18	19,46					
2 years	19	21,00	0,74	0,25	0,06	0,10-0,39	
	20	21,15					
	21	21,38					
	22	22,66					
	23	22,46					
	24	21,10					

Ct mean: threshold cycle mean of 2 replicates;

SD: standard deviation;

Average fold change: average change in expression relative to 30 days group;

SEM: standard error of mean;

CI: confidence interval.

miR-499 (fast-twitch muscle)							
**Group**	**Sample**	**Ct mean**	**SD of 6 replicates**	**Average fold change**	**SEM**	**95% CI (Lower-Upper)**	**F value**
30 days	1	19,53	0,16	1	0,05	0,88-1,12	27,17
	2	19,19					
	3	19,66					
	4	19,46					
	5	19,34					
	6	19,49					
90 days	7	25,48	2,24	0,55	0,17	0,10-1,00	
	8	20,26					
	9	21,69					
	10	21,08					
	11	19,65					
	12	19,39					
150 days	13	25,31	1,41	0,02	0,01	-0,005-0,04	
	14	26,98					
	15	27,63					
	16	23,71					
	17	26,76					
	18	26,26					
2 years	19	25,32	0,96	0,03	0,01	0,005-0,05	
	20	25,97					
	21	25,27					
	22	23,54					
	23	26,10					
	24	24,51					

Ct mean: threshold cycle mean of 2 replicates;
SD: standard deviation;
Average fold change: average change in expression relative to 30 days group;
SEM: standard error of mean;
CI: confidence interval.

sox6 (fast-twitch muscle)							
**Group**	**Sample**	**Ct mean**	**SD of 6 replicates**	**Average fold change**	**SEM**	**95% CI (Lower-Upper)**	**F value**
30 days	1	16,13	0,18	1	0,06	0,85-1,15	84,92
	2	16,25					
	3	16,54					
	4	16,18					
	5	16,55					
	6	16,22					
90 days	7	16,12	0,73	0,90	0,11	0,63-1,18	
	8	16,54					
	9	16,66					
	10	17,72					
	11	16,14					
	12	15,57					
150 days	13	14,43	0,31	4,16	0,42	3,09-5,24	
	14	14,38					
	15	14,27					
	16	14,50					
	17	13,65					
	18	14,20					
2 years	19	13,97	0,34	7,22	0,48	5,97-8,46	
	20	13,19					
	21	13,75					
	22	13,64					
	23	13,09					
	24	13,35					

Ct mean: threshold cycle mean of 2 replicates;
SD: standard deviation;
Average fold change: average change in expression relative to 30 days group;
SEM: standard error of mean;
CI: confidence interval.

myod (fast-twitch muscle)							
**Group**	**Sample**	**Ct mean**	**SD of 6 replicates**	**Average fold change**	**SEM**	**95% CI (Lower-Upper)**	**F value**
30 days	1	16,13	0,46	1	0,10	0,74-1,26	8,47
	2	14,94					
	3	15,56					
	4	14,92					
	5	15,17					
	6	15,40					
90 days	7	16,14	0,41	0,66	0,05	0,52-0,79	
	8	16,10					
	9	15,99					
	10	16,37					
	11	15,55					
	12	15,27					
150 days	13	15,73	0,26	0,83	0,05	0,68-0,97	
	14	15,49					
	15	15,57					
	16	15,50					
	17	15,95					
	18	15,19					
2 years	19	16,84	0,39	0,58	0,02	0,53-0,64	
	20	15,92					
	21	16,07					
	22	15,91					
	23	15,75					
	24	16,29					

Ct mean: threshold cycle mean of 2 replicates;

SD: standard deviation;

Average fold change: average change in expression relative to 30 days group;

SEM: standard error of mean;

CI: confidence interval.

myogenin (fast-twitch muscle)							
**Group**	**Sample**	**Ct mean**	**SD of 6 replicates**	**Average fold change**	**SEM**	**95% CI (Lower-Upper)**	**F value**
30 days	1	16,13	1,91	1	0,44	-0,14-2,14	22,53
	2	18,93					
	3	18,77					
	4	17,06					
	5	21,70					
	6	18,39					
90 days	7	16,34	0,57	1,88	0,14	1,53-2,23	
	8	17,10					
	9	16,86					
	10	17,55					
	11	16,05					
	12	16,28					
150 days	13	16,28	0,28	3,49	0,24	2,86-4,11	
	14	15,57					
	15	15,65					
	16	16,02					
	17	15,69					
	18	15,66					
2 years	19	15,54	0,61	6,07	0,78	4,07-8,07	
	20	15,03					
	21	14,48					
	22	14,90					
	23	14,71					
	24	16,14					

Ct mean: threshold cycle mean of 2 replicates;
SD: standard deviation;
Average fold change: average change in expression relative to 30 days group;
SEM: standard error of mean;
CI: confidence interval.

miR-1 (slow-twitch muscle)							
**Group**	**Sample**	**Ct mean**	**SD of 6 replicates**	**Average fold change**	**SEM**	**95% CI (Lower-Upper)**	**F value**
150 days	13	21,07	0,38	1	0,10	0,74-1,26	3,45
	14	20,77					
	15	20,67					
	16	19,96					
	17	20,82					
	18	20,46					
2 years	19	18,68	0,15	4,19	0,19	3,71-4,68	
	20	18,28					
	21	18,52					
	22	18,40					
	23	18,40					
	24	18,61					

Ct mean: threshold cycle mean of 2 replicates;
SD: standard deviation;
Average fold change: average change in expression relative to 150 days group;
SEM: standard error of mean;
CI: confidence interval.

hdac4 (slow-twitch muscle)							
**Group**	**Sample**	**Ct mean**	**SD of 6 replicates**	**Average fold change**	**SEM**	**95% CI (Lower-Upper)**	**F value**
150 days	13	16,13	0,31	1	0,16	0,60-1,41	2,43
	14	15,69					
	15	15,83					
	16	15,17					
	17	15,61					
	18	15,69					
2 years	19	16,36	0,75	0,50	0,10	0,24-0,76	
	20	17,25					
	21	17,52					
	22	17,63					
	23	15,81					
	24	16,33					

Ct mean: threshold cycle mean of 2 replicates;
SD: standard deviation;
Average fold change: average change in expression relative to 150 days group;
SEM: standard error of mean;
CI: confidence interval.

miR-133a (slow-twitch muscle)							
**Group**	**Sample**	**Ct mean**	**SD of 6 replicates**	**Average fold change**	**SEM**	**95% CI (Lower-Upper)**	**F value**
150 days	13	21,07	0,35	1	0,10	0,74-1,26	3,82
	14	20,82					
	15	20,97					
	16	20,38					
	17	20,80					
	18	20,17					
2 years	19	20,68	0,13	1,03	0,05	0,89-1,16	
	20	20,74					
	21	20,45					
	22	20,67					
	23	20,45					
	24	20,56					

Ct mean: threshold cycle mean of 2 replicates;
SD: standard deviation;
Average fold change: average change in expression relative to 150 days group;
SEM: standard error of mean;
CI: confidence interval.

miR-133b (slow-twitch muscle)							
**Group**	**Sample**	**Ct mean**	**SD of 6 replicates**	**Average fold change**	**SEM**	**95% CI (Lower-Upper)**	**F value**
150 days	13	21,07	0,51	1	0,14	0,65-1,35	19,14
	14	20,75					
	15	20,97					
	16	19,78					
	17	20,38					
	18	20,08					
2 years	19	20,65	0,09	0,85	0,03	0,77-0,93	
	20	20,67					
	21	20,47					
	22	20,68					
	23	20,69					
	24	20,57					

Ct mean: threshold cycle mean of 2 replicates;
SD: standard deviation;
Average fold change: average change in expression relative to 150 days group;
SEM: standard error of mean;
CI: confidence interval.

<table>
<tr><th colspan="8">srf (slow-twitch muscle)</th></tr>
<tr><th>Group</th><th>Sample</th><th>Ct mean</th><th>SD of 6 replicates</th><th>Average fold change</th><th>SEM</th><th>95% CI (Lower-Upper)</th><th>F value</th></tr>
<tr><td rowspan="6">150 days</td><td>13</td><td>16,13</td><td rowspan="6">0,81</td><td rowspan="6">1</td><td rowspan="6">0,23</td><td rowspan="6">0,42-1,58</td><td rowspan="12">5,46</td></tr>
<tr><td>14</td><td>15,76</td></tr>
<tr><td>15</td><td>17,40</td></tr>
<tr><td>16</td><td>16,89</td></tr>
<tr><td>17</td><td>17,85</td></tr>
<tr><td>18</td><td>17,41</td></tr>
<tr><td rowspan="6">2 years</td><td>19</td><td>17,34</td><td rowspan="6">0,40</td><td rowspan="6">0,73</td><td rowspan="6">0,10</td><td rowspan="6">0,48-0,97</td></tr>
<tr><td>20</td><td>17,15</td></tr>
<tr><td>21</td><td>17,62</td></tr>
<tr><td>22</td><td>17,70</td></tr>
<tr><td>23</td><td>16,56</td></tr>
<tr><td>24</td><td>17,25</td></tr>
</table>

Ct mean: threshold cycle mean of 2 replicates;

SD: standard deviation;

Average fold change: average change in expression relative to 150 days group;

SEM: standard error of mean;

CI: confidence interval.

<table>
<tr><th colspan="8">miR-206 (slow-twitch muscle)</th></tr>
<tr><th>Group</th><th>Sample</th><th>Ct mean</th><th>SD of 6 replicates</th><th>Average fold change</th><th>SEM</th><th>95% CI (Lower-Upper)</th><th>F value</th></tr>
<tr><td rowspan="6">150 days</td><td>13</td><td>20,97</td><td rowspan="6">0,44</td><td rowspan="6">1</td><td rowspan="6">0,12</td><td rowspan="6">0,69-1,31</td><td rowspan="12">2,17</td></tr>
<tr><td>14</td><td>20,88</td></tr>
<tr><td>15</td><td>20,64</td></tr>
<tr><td>16</td><td>19,86</td></tr>
<tr><td>17</td><td>20,28</td></tr>
<tr><td>18</td><td>20,14</td></tr>
<tr><td rowspan="6">2 years</td><td>19</td><td>19,29</td><td rowspan="6">0,33</td><td rowspan="6">1,84</td><td rowspan="6">0,18</td><td rowspan="6">1,38-2,30</td></tr>
<tr><td>20</td><td>19,88</td></tr>
<tr><td>21</td><td>19,12</td></tr>
<tr><td>22</td><td>19,77</td></tr>
<tr><td>23</td><td>19,77</td></tr>
<tr><td>24</td><td>19,25</td></tr>
</table>

Ct mean: threshold cycle mean of 2 replicates;

SD: standard deviation;

Average fold change: average change in expression relative to 150 days group;

SEM: standard error of mean;

CI: confidence interval.

<table>
<tr><th colspan="8">pax7 (slow-twitch muscle)</th></tr>
<tr><th>Group</th><th>Sample</th><th>Ct mean</th><th>SD of 6 replicates</th><th>Average fold change</th><th>SEM</th><th>95% CI (Lower-Upper)</th><th>F value</th></tr>
<tr><td rowspan="6">150 days</td><td>13</td><td>15,98</td><td rowspan="6">0,23</td><td rowspan="6">1</td><td rowspan="6">0,13</td><td rowspan="6">0,66-1,34</td><td rowspan="12">1,10</td></tr>
<tr><td>14</td><td>15,78</td></tr>
<tr><td>15</td><td>15,71</td></tr>
<tr><td>16</td><td>15,37</td></tr>
<tr><td>17</td><td>15,46</td></tr>
<tr><td>18</td><td>15,85</td></tr>
<tr><td rowspan="6">2 years</td><td>19</td><td>16,14</td><td rowspan="6">0,83</td><td rowspan="6">0,57</td><td rowspan="6">0,13</td><td rowspan="6">0,25-0,90</td></tr>
<tr><td>20</td><td>17,30</td></tr>
<tr><td>21</td><td>17,38</td></tr>
<tr><td>22</td><td>17,53</td></tr>
<tr><td>23</td><td>15,66</td></tr>
<tr><td>24</td><td>15,96</td></tr>
</table>

Ct mean: threshold cycle mean of 2 replicates;
SD: standard deviation;
Average fold change: average change in expression relative to 150 days group;
SEM: standard error of mean;
CI: confidence interval.

<table>
<tr><th colspan="8">miR-499 (slow-twitch muscle)</th></tr>
<tr><th>Group</th><th>Sample</th><th>Ct mean</th><th>SD of 6 replicates</th><th>Average fold change</th><th>SEM</th><th>95% CI (Lower-Upper)</th><th>F value</th></tr>
<tr><td rowspan="6">150 days</td><td>13</td><td>21,07</td><td rowspan="6">0,48</td><td rowspan="6">1</td><td rowspan="6">0,13</td><td rowspan="6">0,66-1,34</td><td rowspan="12">3,74</td></tr>
<tr><td>14</td><td>20,59</td></tr>
<tr><td>15</td><td>20,47</td></tr>
<tr><td>16</td><td>19,93</td></tr>
<tr><td>17</td><td>20,00</td></tr>
<tr><td>18</td><td>19,83</td></tr>
<tr><td rowspan="6">2 years</td><td>19</td><td>20,38</td><td rowspan="6">0,33</td><td rowspan="6">1,14</td><td rowspan="6">0,07</td><td rowspan="6">0,96-1,32</td></tr>
<tr><td>20</td><td>19,98</td></tr>
<tr><td>21</td><td>19,44</td></tr>
<tr><td>22</td><td>20,18</td></tr>
<tr><td>23</td><td>20,22</td></tr>
<tr><td>24</td><td>19,90</td></tr>
</table>

Ct mean: threshold cycle mean of 2 replicates;
SD: standard deviation;
Average fold change: average change in expression relative to 150 days group;
SEM: standard error of mean;
CI: confidence interval.

***sox6* (slow-twitch muscle)**							
**Group**	**Sample**	**Ct mean**	**SD of 6 replicates**	**Average fold change**	**SEM**	**95% CI (Lower-Upper)**	**F value**
150 days	13	16,13	0,30	1	0,04	0,88-1,12	4,50
	14	15,78					
	15	15,98					
	16	16,60					
	17	15,93					
	18	15,84					
2 years	19	16,54	0,85	0,38	0,10	0,14-0,63	
	20	17,05					
	21	18,55					
	22	18,50					
	23	18,28					
	24	17,31					

Ct mean: threshold cycle mean of 2 replicates;
SD: standard deviation;
Average fold change: average change in expression relative to 150 days group;
SEM: standard error of mean;
CI: confidence interval.

***myod* (slow-twitch muscle)**							
**Group**	**Sample**	**Ct mean**	**SD of 6 replicates**	**Average fold change**	**SEM**	**95% CI (Lower-Upper)**	**F value**
150 days	13	16,06	0,30	1	0,14	0,64-1,36	1,86
	14	15,78					
	15	15,79					
	16	15,21					
	17	15,52					
	18	15,47					
2 years	19	15,93	0,34	0,82	0,10	0,56-1,09	
	20	15,52					
	21	16,12					
	22	16,05					
	23	15,61					
	24	16,44					

Ct mean: threshold cycle mean of 2 replicates;
SD: standard deviation;
Average fold change: average change in expression relative to 150 days group;
SEM: standard error of mean;
CI: confidence interval.

<table>
<tr><th colspan="8">myogenin (slow-twitch muscle)</th></tr>
<tr><th>Group</th><th>Sample</th><th>Ct mean</th><th>SD of 6 replicates</th><th>Average fold change</th><th>SEM</th><th>95% CI (Lower-Upper)</th><th>F value</th></tr>
<tr><td rowspan="6">150 days</td><td>13</td><td>16,08</td><td rowspan="6">0,27</td><td rowspan="6">1</td><td rowspan="6">0,12</td><td rowspan="6">0,70-1,30</td><td rowspan="12">231,44</td></tr>
<tr><td>14</td><td>15,78</td></tr>
<tr><td>15</td><td>15,85</td></tr>
<tr><td>16</td><td>15,63</td></tr>
<tr><td>17</td><td>15,26</td></tr>
<tr><td>18</td><td>15,65</td></tr>
<tr><td rowspan="6">2 years</td><td>19</td><td>13,88</td><td rowspan="6">0,84</td><td rowspan="6">7,70</td><td rowspan="6">1,75</td><td rowspan="6">3,19-12,21</td></tr>
<tr><td>20</td><td>13,14</td></tr>
<tr><td>21</td><td>13,88</td></tr>
<tr><td>22</td><td>12,78</td></tr>
<tr><td>23</td><td>12,06</td></tr>
<tr><td>24</td><td>11,99</td></tr>
</table>

Ct mean: threshold cycle mean of 2 replicates;

SD: standard deviation;

Average fold change: average change in expression relative to 150 days group;

SEM: standard error of mean;

CI: confidence interval.

miR-1 (myoblast cell culture)							
**Group**	**Sample**	**Ct mean**	**SD of 3 replicates**	**Average fold change**	**SEM**	**95% CI (Lower-Upper)**	**F value**
2-4 days	25	20,80	0,19	1	0,09	0,62-1,38	9,02
	26	20,80					
	27	20,48					
5-8 days	28	19,51	0,40	1,76	0,14	1,14-2,38	
	29	20,13					
	30	20,25					
9-12 days	31	18,65	0,38	3,29	0,65	0,49-6,09	
	32	19,32					
	33	19,31					

Ct mean: threshold cycle mean of 2 replicates;
SD: standard deviation;
Average fold change: average change in expression relative to 2-4 days group;
SEM: standard error of mean;
CI: confidence interval.

hdac4 (myoblast cell culture)							
**Group**	**Sample**	**Ct mean**	**SD of 3 replicates**	**Average fold change**	**SEM**	**95% CI (Lower-Upper)**	**F value**
2-4 days	25	13,96	0,43	1	0,19	0,18-1,82	8,55
	26	14,82					
	27	14,29					
5-8 days	28	14,27	0,32	1,13	0,03	0,98-1,28	
	29	14,40					
	30	13,80					
9-12 days	31	15,13	0,03	0,51	0,02	0,42-0,60	
	32	15,18					
	33	15,11					

Ct mean: threshold cycle mean of 2 replicates;
SD: standard deviation;
Average fold change: average change in expression relative to 2-4 days group;
SEM: standard error of mean;
CI: confidence interval.

miR-133a (myoblast cell culture)							
**Group**	**Sample**	**Ct mean**	**SD of 3 replicates**	**Average fold change**	**SEM**	**95% CI (Lower-Upper)**	**F value**
2-4 days	25	20,32	0,47	1	0,14	0,41-1,59	7,33
	26	19,40					
	27	19,72					
5-8 days	28	19,51	0,17	1,41	0,21	0,49-2,32	
	29	19,22					
	30	19,51					
9-12 days	31	20,98	0,36	0,60	0,06	0,36-0,83	
	32	20,62					
	33	20,26					

Ct mean: threshold cycle mean of 2 replicates;
SD: standard deviation;
Average fold change: average change in expression relative to 2-4 days group;
SEM: standard error of mean;
CI: confidence interval.

miR-133b (myoblast cell culture)							
**Group**	**Sample**	**Ct mean**	**SD of 3 replicates**	**Average fold change**	**SEM**	**95% CI (Lower-Upper)**	**F value**
2-4 days	25	20,25	0,36	1	0,10	0,58-1,42	10,99
	26	19,53					
	27	19,88					
5-8 days	28	19,75	0,14	1,29	0,16	0,60-1,97	
	29	19,65					
	30	19,47					
9-12 days	31	21,11	0,33	0,55	0,04	0,37-0,74	
	32	20,85					
	33	20,46					

Ct mean: threshold cycle mean of 2 replicates;
SD: standard deviation;
Average fold change: average change in expression relative to 2-4 days group;
SEM: standard error of mean;
CI: confidence interval.

srf (myoblast cell culture)							
**Group**	**Sample**	**Ct mean**	**SD of 3 replicates**	**Average fold change**	**SEM**	**95% CI (Lower-Upper)**	**F value**
2-4 days	25	16,26	0,26	1	0,14	0,41-1,59	14,79
	26	16,79					
	27	16,51					
5-8 days	28	18,25	0,50	0,42	0,12	-0,09-0,93	
	29	17,30					
	30	18,08					
9-12 days	31	16,25	0,14	1,20	0,02	1,11-1,30	
	32	16,01					
	33	16,00					

Ct mean: threshold cycle mean of 2 replicates;
SD: standard deviation;
Average fold change: average change in expression relative to 2-4 days group;
SEM: standard error of mean;
CI: confidence interval.

miR-206 (myoblast cell culture)							
**Group**	**Sample**	**Ct mean**	**SD of 3 replicates**	**Average fold change**	**SEM**	**95% CI (Lower-Upper)**	**F value**
2-4 days	25	20,57	0,07	1	0,06	0,74-1,26	30,19
	26	20,61					
	27	20,70					
5-8 days	28	18,57	0,15	4,15	0,41	2,40-5,91	
	29	18,60					
	30	18,84					
9-12 days	31	18,70	0,41	5,13	0,54	2,80-7,46	
	32	18,46					
	33	17,89					

Ct mean: threshold cycle mean of 2 replicates;
SD: standard deviation;
Average fold change: average change in expression relative to 2-4 days group;
SEM: standard error of mean;
CI: confidence interval.

pax7 (myoblast cell culture)							
**Group**	**Sample**	**Ct mean**	**SD of 3 replicates**	**Average fold change**	**SEM**	**95% CI (Lower-Upper)**	**F value**
2-4 days	25	12,87	0,12	1	0,11	0,51-1,50	28,00
	26	13,09					
	27	12,90					
5-8 days	28	13,67	0,36	0,66	0,03	0,51-0,81	
	29	13,88					
	30	13,17					
9-12 days	31	15,50	0,51	0,23	0,04	0,08-0,39	
	32	14,75					
	33	14,52					

Ct mean: threshold cycle mean of 2 replicates;
SD: standard deviation;
Average fold change: average change in expression relative to 2-4 days group;
SEM: standard error of mean;
CI: confidence interval.

miR-499 (myoblast cell culture)							
**Group**	**Sample**	**Ct mean**	**SD of 3 replicates**	**Average fold change**	**SEM**	**95% CI (Lower-Upper)**	**F value**
2-4 days	25	20,15	0,55	1	0,16	0,31-1,69	5,78
	26	19,11					
	27	19,35					
5-8 days	28	18,19	0,33	3,25	0,71	0,19-6,32	
	29	17,58					
	30	18,08					
9-12 days	31	18,35	0,61	1,70	0,40	-0,006-3,41	
	32	19,54					
	33	18,74					

Ct mean: threshold cycle mean of 2 replicates;
SD: standard deviation;
Average fold change: average change in expression relative to 2-4 days group;
SEM: standard error of mean;
CI: confidence interval.

<table>
<tr><th colspan="8">sox6 (myoblast cell culture)</th></tr>
<tr><th>Group</th><th>Sample</th><th>Ct mean</th><th>SD of 3 replicates</th><th>Average fold change</th><th>SEM</th><th>95% CI (Lower-Upper)</th><th>F value</th></tr>
<tr><td rowspan="3">2-4 days</td><td>25</td><td>14,94</td><td rowspan="3">0,69</td><td rowspan="3">1</td><td rowspan="3">0,24</td><td rowspan="3">-0,03-2,03</td><td rowspan="9">0,13</td></tr>
<tr><td>26</td><td>13,61</td></tr>
<tr><td>27</td><td>14,58</td></tr>
<tr><td rowspan="3">5-8 days</td><td>28</td><td>14,67</td><td rowspan="3">0,75</td><td rowspan="3">1,04</td><td rowspan="3">0,23</td><td rowspan="3">0,06-2,03</td></tr>
<tr><td>29</td><td>14,86</td></tr>
<tr><td>30</td><td>13,47</td></tr>
<tr><td rowspan="3">9-12 days</td><td>31</td><td>13,97</td><td rowspan="3">0,16</td><td rowspan="3">1,14</td><td rowspan="3">0,08</td><td rowspan="3">0,80-1,48</td></tr>
<tr><td>32</td><td>14,14</td></tr>
<tr><td>33</td><td>13,82</td></tr>
</table>

Ct mean: threshold cycle mean of 2 replicates;
SD: standard deviation;
Average fold change: average change in expression relative to 2-4 days group;
SEM: standard error of mean;
CI: confidence interval.

<table>
<tr><th colspan="8">myod (myoblast cell culture)</th></tr>
<tr><th>Group</th><th>Sample</th><th>Ct mean</th><th>SD of 3 replicates</th><th>Average fold change</th><th>SEM</th><th>95% CI (Lower-Upper)</th><th>F value</th></tr>
<tr><td rowspan="3">2-4 days</td><td>25</td><td>13,92</td><td rowspan="3">0,31</td><td rowspan="3">1</td><td rowspan="3">0,20</td><td rowspan="3">0,15-1,85</td><td rowspan="9">6,01</td></tr>
<tr><td>26</td><td>13,55</td></tr>
<tr><td>27</td><td>13,29</td></tr>
<tr><td rowspan="3">5-8 days</td><td>28</td><td>14,45</td><td rowspan="3">0,26</td><td rowspan="3">0,62</td><td rowspan="3">0,02</td><td rowspan="3">0,54-0,71</td></tr>
<tr><td>29</td><td>14,35</td></tr>
<tr><td>30</td><td>13,96</td></tr>
<tr><td rowspan="3">9-12 days</td><td>31</td><td>14,46</td><td rowspan="3">0,22</td><td rowspan="3">0,43</td><td rowspan="3">0,05</td><td rowspan="3">0,21-0,65</td></tr>
<tr><td>32</td><td>14,58</td></tr>
<tr><td>33</td><td>14,89</td></tr>
</table>

Ct mean: threshold cycle mean of 2 replicates;
SD: standard deviation;
Average fold change: average change in expression relative to 2-4 days group;
SEM: standard error of mean;
CI: confidence interval.

myogenin (myoblast cell culture)							
**Group**	**Sample**	**Ct mean**	**SD of 3 replicates**	**Average fold change**	**SEM**	**95% CI (Lower-Upper)**	**F value**
2-4 days	25	15,47	0,18	1	0,16	0,30-1,70	474,70
	26	15,40					
	27	15,12					
5-8 days	28	14,19	0,24	2,35	0,02	2,26-2,44	
	29	14,29					
	30	13,83					
9-12 days	31	11,72	0,01	10,99	0,40	9,27-12,70	
	32	11,69					
	33	11,71					

Ct mean: threshold cycle mean of 2 replicates;
SD: standard deviation;
Average fold change: average change in expression relative to 2-4 days group;
SEM: standard error of mean;
CI: confidence interval.

Reference genes			
**Group**	**Sample**	**Ct mean *U6 snRNA***	**Ct mean *18S rRNA***
30 days (fast-twitch muscle)	1	15,47	10,53
	2	15,29	10,18
	3	15,52	10,68
	4	15,14	10,50
	5	15,34	10,43
	6	15,23	10,43
90 days (fast-twitch muscle)	7	15,48	10,14
	8	15,55	10,57
	9	15,34	10,74
	10	15,45	10,85
	11	15,45	10,05
	12	15,43	10,13
150 days (fast-twitch muscle)	13	15,40	10,44
	14	15,44	10,27
	15	15,49	10,59
	16	15,52	10,62
	17	15,48	10,42
	18	15,62	10,21
2 years (fast-twitch muscle)	19	15,39	10,99
	20	15,55	10,45
	21	15,39	10,49
	22	15,44	10,32
	23	15,65	10,16
	24	15,38	10,57
150 days (slow-twitch muscle)	13	15,38	10,63
	14	15,19	10,24
	15	15,61	10,51
	16	15,24	10,84
	17	15,56	10,56
	18	15,43	10,08
2 years (slow-twitch muscle)	19	15,59	10,46
	20	15,25	10,77
	21	15,15	10,50
	22	15,35	10,32
	23	15,46	10,46
	24	15,33	10,58
2-4 days (myoblast cell culture)	25	15,72	10,24
	26	15,19	10,64
	27	15,32	10,16
5-8 days (myoblast cell culture)	28	15,14	10,52
	29	15,05	10,96
	30	15,64	10,51
9-12 days (myoblast cell culture)	31	15,17	10,46
	32	15,17	10,84
	33	15,42	10,70

Ct mean: threshold cycle mean of 2 replicates.
