# Supplementary figures and images for: Differential microRNA Expression in Fast- and Slow-Twitch Skeletal Muscle of Piaractus mesopotamicus during Growth
Source: PLoS One. 2015 Nov 3;10(11):e0141967. doi: 10.1371/journal.pone.0141967 (PMC4631509; doi:10.1371/journal.pone.0141967)

# Reference Genes

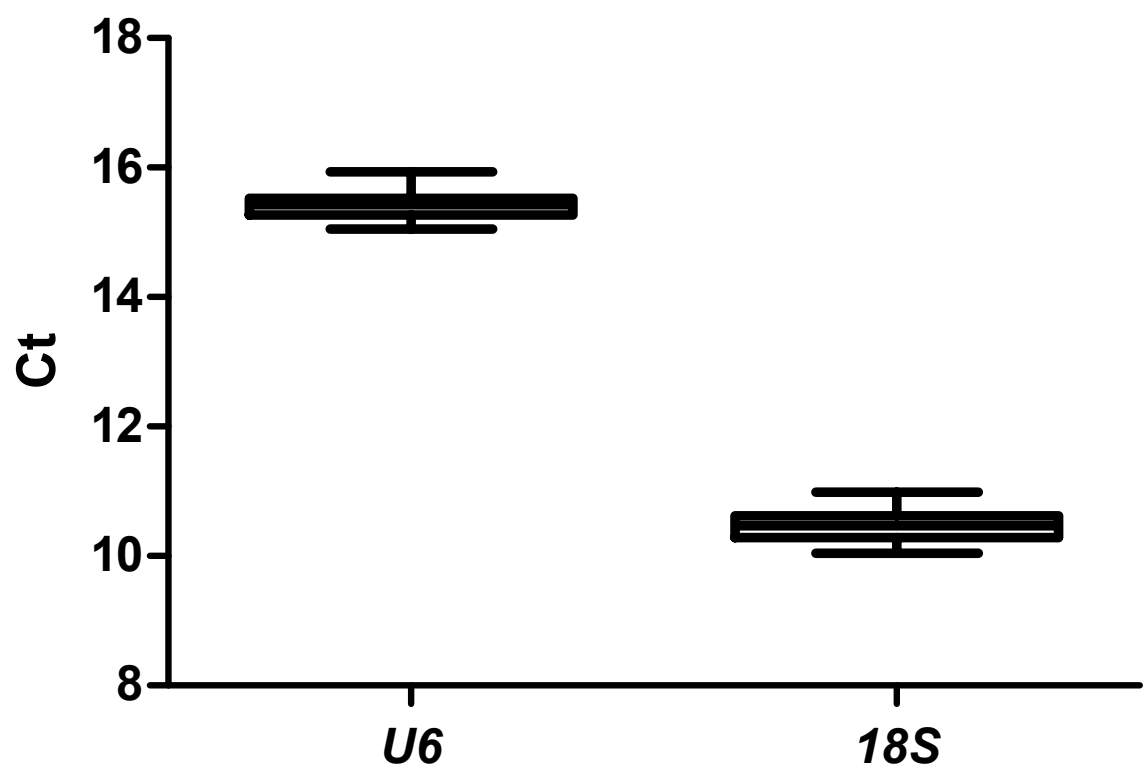

Supplement: S2 File — The Ct (threshold cycle) values of the U6 snRNA and 18S rRNA were similar between all groups. (PDF) [file pone.0141967.s002.pdf]
