# Supplementary material for: Differential microRNA Expression in Fast- and Slow-Twitch Skeletal Muscle of Piaractus mesopotamicus during Growth
Source: PLoS One. 2015 Nov 3;10(11):e0141967. doi: 10.1371/journal.pone.0141967 (PMC4631509; doi:10.1371/journal.pone.0141967)
Supplement: S3 File — The seed-matched sequences at the 3’UTR of the mRNAs are shown in red, the conserved regions between the aligned sequences are indicated by stars, and the dashes represent the manually inserted gaps. (PDF) [file pone.0141967.s003.pdf]

hdac4

```
dre-miR-1          3' U A U G U A U G A A G A A A U G U A A G G U  5'
                                |       | |       | | | | | | |
H. sapiens         5' T C T T T T T G A T C A G A A C A T T C C T  3'
M. musculus        5' T T T C T T T C C T C A G A A C A T T C C T  3'
Danio rerio        5' A A C T T T C G A G C A G G A C A T A G A C  3'
P. mesopotamicus   5' T G C T T T C G A A C G G G A C A T G G A C  3'
                              * *         *   *   * * * *
```

srf

```
dre-miR-133a-3p    3' G U C G A C C A A C U U C C C C U G G U U U  5'
                        |       | | | | | |   | |   | | | | | | |
H. sapiens         5' G A G A G G G A A G A G G G A G A C C A A A  3'
M. musculus        5' A A A A G G G A A G A T G G A G A C C A A A  3'
Danio rerio        5' C C - - - T C A A T A T G A A G T T A A A A  3'
P. mesopotamicus   5' G T C G G T G A A A A T C T G G A T T A A A  3'
                                    * *   *         *       * * *
```

srf

dre-miR-133b-3p	3'	A	U	C	G	A	C	C	A	A	C	U	U	C	C	C	C	U	G	G	U	U	U	5'
			|				|	|	|	|	|	|		|	|		|	|	|	|	|	|	|	
H. sapiens	5'	G	A	G	A	G	G	G	A	A	G	A	G	G	G	A	G	A	C	C	A	A	A	3'
M. musculus	5'	A	A	A	A	G	G	G	A	A	G	A	T	G	G	A	G	A	C	C	A	A	A	3'
Danio rerio	5'	C	C	−	−	−	T	C	A	A	T	A	T	G	A	A	G	T	T	A	A	A	A	3'
P. mesopotamicus	5'	G	T	C	G	G	T	G	A	A	A	A	T	C	T	G	G	A	T	T	A	A	A	3'
									*	*		*					*				*	*	*	

pax7

dre-miR-206-3p	3'	G	G	U	G	U	G	U	G	A	A	G	G	A	A	U	G	U	A	A	G	G	U	5'
					|		|	|								|	|	|	|	|	|	|		
H. sapiens	5'	A	T	C	C	C	C	A	G	G	A	G	G	C	G	A	C	A	T	T	C	C	T	3'
Danio rerio	5'	C	T	A	T	A	T	C	C	G	A	T	T	G	C	C	C	A	T	T	C	C	A	3'
P. mesopotamicus	5'	C	C	C	T	T	T	C	C	A	G	T	T	G	C	C	C	A	T	T	C	T	T	3'
																	*	*	*	*	*			

sox6

dre-miR-499-5p	3'	A	−	U	U	U	G	U	A	G	U	G	A	C	G	U	U	C	A	G	A	A	U	U	5'
		|		|	|			|					|	|		|	|	|	|	|	|	|	|		
H. sapiens	5'	T	−	A	A	−	−	T	A	T	T	T	T	G	A	A	A	G	T	C	T	T	A	T	3'
Danio rerio	5'	T	A	A	−	−	−	C	C	T	A	T	A	A	A	A	A	G	T	C	G	T	G	A	3'
P. mesopotamicus	5'	T	G	A	A	C	T	C	C	T	T	C	T	A	A	G	A	G	T	T	T	T	A	G	3'
		*		*						*					*		*	*	*			*			
