# Supplementary material for: Differential microRNA Expression in Fast- and Slow-Twitch Skeletal Muscle of Piaractus mesopotamicus during Growth
Source: PLoS One. 2015 Nov 3;10(11):e0141967. doi: 10.1371/journal.pone.0141967 (PMC4631509; doi:10.1371/journal.pone.0141967)
Supplement: S4 File — The seed sequences of the miRNAs are in the purple box, and the fish species are in the gray box. (PDF) [file pone.0141967.s004.pdf]

miR-1

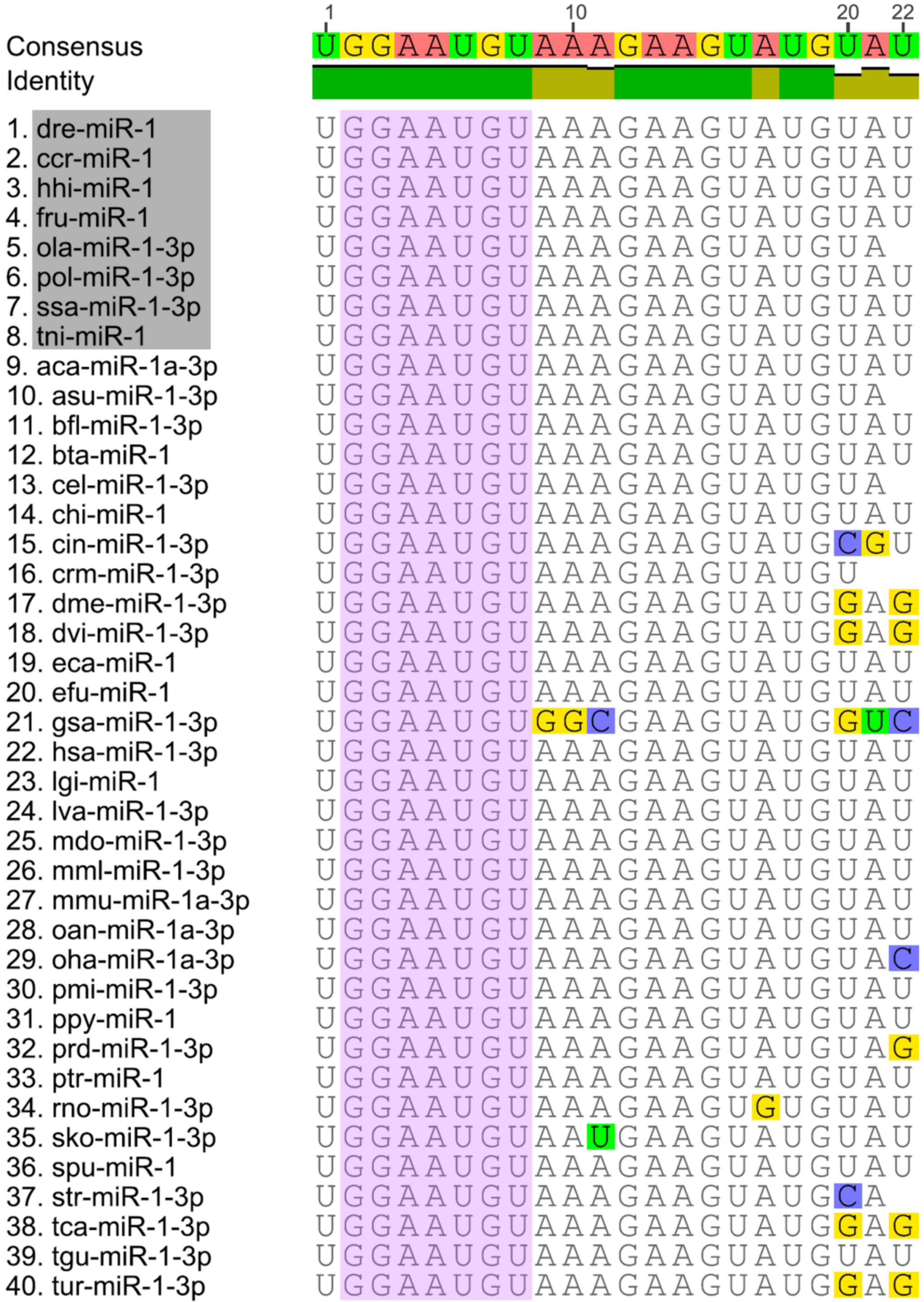
1 10 20 22
Consensus UGGAAUGUAAAGAAGUAUGUAU
Identity
1. dre-miR-1 UGGAAUGUAAAGAAGUAUGUAU
2. ccr-miR-1 UGGAAUGUAAAGAAGUAUGUAU
3. hhi-miR-1 UGGAAUGUAAAGAAGUAUGUAU
4. fru-miR-1 UGGAAUGUAAAGAAGUAUGUAU
5. ola-miR-1-3p UGGAAUGUAAAGAAGUAUGUA
6. pol-miR-1-3p UGGAAUGUAAAGAAGUAUGUAU
7. ssa-miR-1-3p UGGAAUGUAAAGAAGUAUGUAU
8. tni-miR-1 UGGAAUGUAAAGAAGUAUGUAU
9. aca-miR-1a-3p UGGAAUGUAAAGAAGUAUGUAU
10. asu-miR-1-3p UGGAAUGUAAAGAAGUAUGUA
11. bfl-miR-1-3p UGGAAUGUAAAGAAGUAUGUAU
12. bta-miR-1 UGGAAUGUAAAGAAGUAUGUAU
13. cel-miR-1-3p UGGAAUGUAAAGAAGUAUGUA
14. chi-miR-1 UGGAAUGUAAAGAAGUAUGUAU
15. cin-miR-1-3p UGGAAUGUAAAGAAGUAUGCGU
16. crm-miR-1-3p UGGAAUGUAAAGAAGUAUGU
17. dme-miR-1-3p UGGAAUGUAAAGAAGUAUGGAG
18. dvi-miR-1-3p UGGAAUGUAAAGAAGUAUGGAG
19. eca-miR-1 UGGAAUGUAAAGAAGUAUGUAU
20. efu-miR-1 UGGAAUGUAAAGAAGUAUGUAU
21. gsa-miR-1-3p UGGAAUGUGGCGAAGUAUGGUC
22. hsa-miR-1-3p UGGAAUGUAAAGAAGUAUGUAU
23. lgi-miR-1 UGGAAUGUAAAGAAGUAUGUAU
24. lva-miR-1-3p UGGAAUGUAAAGAAGUAUGUAU
25. mdo-miR-1-3p UGGAAUGUAAAGAAGUAUGUAU
26. mml-miR-1-3p UGGAAUGUAAAGAAGUAUGUAU
27. mmu-miR-1a-3p UGGAAUGUAAAGAAGUAUGUAU
28. oan-miR-1a-3p UGGAAUGUAAAGAAGUAUGUAU
29. oha-miR-1a-3p UGGAAUGUAAAGAAGUAUGUAC
30. pmi-miR-1-3p UGGAAUGUAAAGAAGUAUGUAU
31. ppy-miR-1 UGGAAUGUAAAGAAGUAUGUAU
32. prd-miR-1-3p UGGAAUGUAAAGAAGUAUGUAG
33. ptr-miR-1 UGGAAUGUAAAGAAGUAUGUAU
34. rno-miR-1-3p UGGAAUGUAAAGAAGUGUGUAU
35. sko-miR-1-3p UGGAAUGUAAUGAAGUAUGUAU
36. spu-miR-1 UGGAAUGUAAAGAAGUAUGUAU
37. str-miR-1-3p UGGAAUGUAAAGAAGUAUGCA
38. tca-miR-1-3p UGGAAUGUAAAGAAGUAUGGAG
39. tgu-miR-1-3p UGGAAUGUAAAGAAGUAUGUAU
40. tur-miR-1-3p UGGAAUGUAAAGAAGUAUGGAG

miR-133a-3p

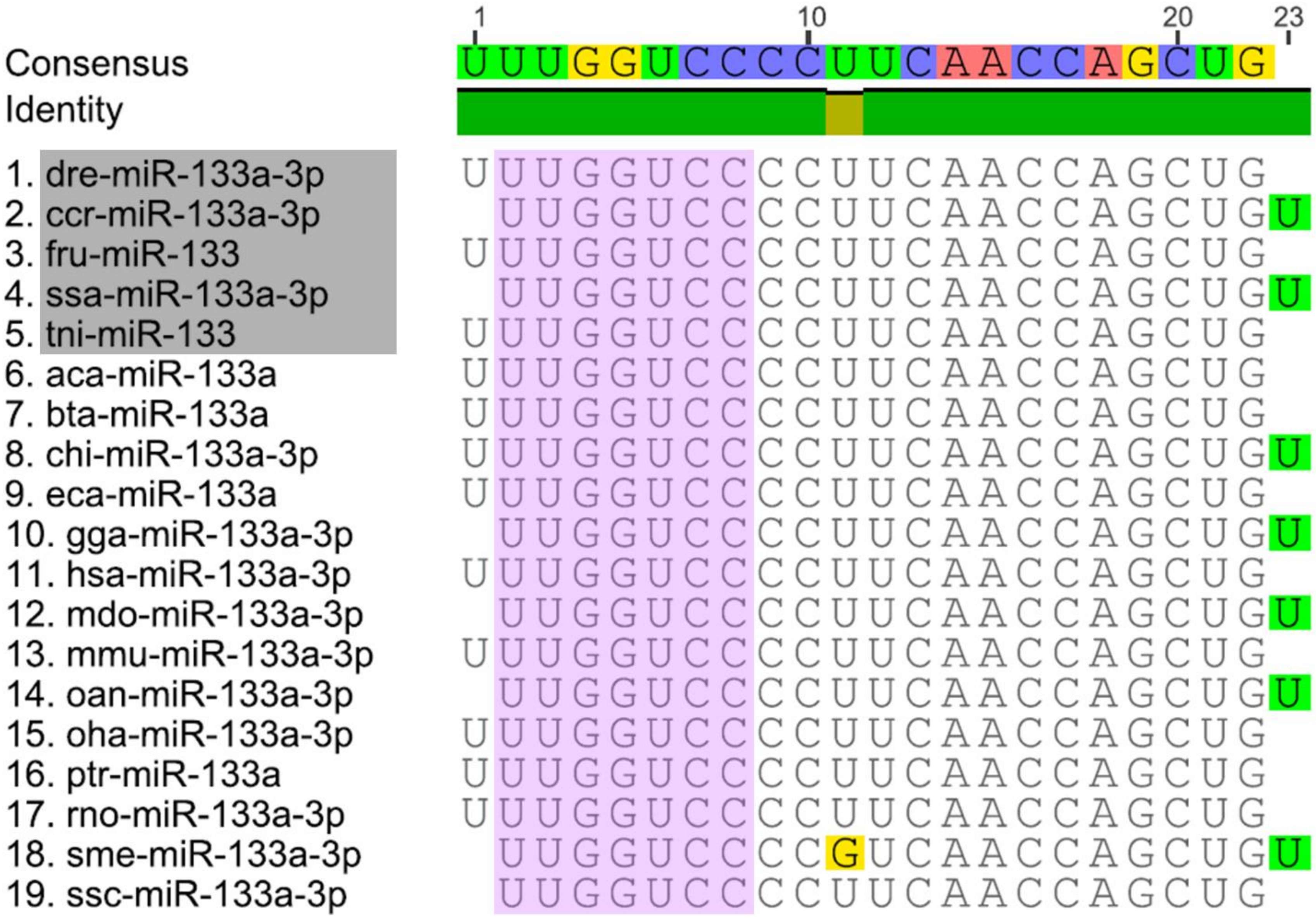

1
10
20
23
Consensus
UUUGGUCCCCUUCAACCAGCUG
Identity
1. dre-miR-133a-3p UUUGGUCCCCUUCAACCAGCUG
2. ccr-miR-133a-3p UUGGUCCCCUUCAACCAGCUGU
3. fru-miR-133 UUUGGUCCCCUUCAACCAGCUG
4. ssa-miR-133a-3p UUGGUCCCCUUCAACCAGCUGU
5. tni-miR-133 UUUGGUCCCCUUCAACCAGCUG
6. aca-miR-133a UUUGGUCCCCUUCAACCAGCUG
7. bta-miR-133a UUUGGUCCCCUUCAACCAGCUG
8. chi-miR-133a-3p UUUGGUCCCCUUCAACCAGCUGU
9. eca-miR-133a UUUGGUCCCCUUCAACCAGCUG
10. gga-miR-133a-3p UUGGUCCCCUUCAACCAGCUGU
11. hsa-miR-133a-3p UUUGGUCCCCUUCAACCAGCUG
12. mdo-miR-133a-3p UUGGUCCCCUUCAACCAGCUGU
13. mmu-miR-133a-3p UUUGGUCCCCUUCAACCAGCUG
14. oan-miR-133a-3p UUGGUCCCCUUCAACCAGCUGU
15. oha-miR-133a-3p UUUGGUCCCCUUCAACCAGCUG
16. ptr-miR-133a UUUGGUCCCCUUCAACCAGCUG
17. rno-miR-133a-3p UUUGGUCCCCUUCAACCAGCUG
18. sme-miR-133a-3p UUGGUCCCCGUCAACCAGCUGU
19. ssc-miR-133a-3p UUGGUCCCCUUCAACCAGCUG

miR-133b-3p

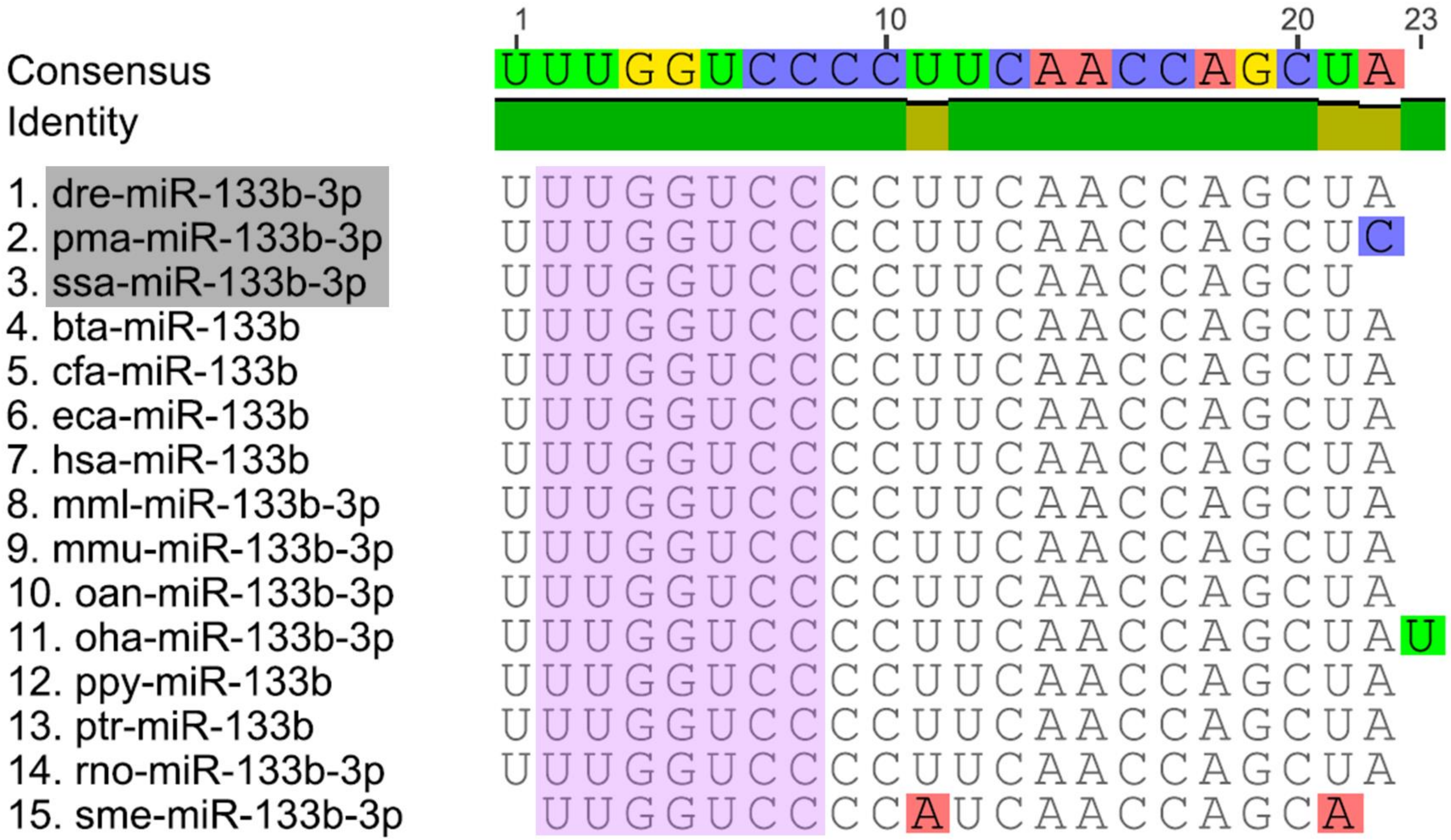

1
10
20
23
Consensus UUUGGUCCCCUUCAACCAGCUA
Identity
1. dre-miR-133b-3p UUUGGUCCCCUUCAACCAGCUA
2. pma-miR-133b-3p UUUGGUCCCCUUCAACCAGCUC
3. ssa-miR-133b-3p UUUGGUCCCCUUCAACCAGCU
4. bta-miR-133b UUUGGUCCCCUUCAACCAGCUA
5. cfa-miR-133b UUUGGUCCCCUUCAACCAGCUA
6. eca-miR-133b UUUGGUCCCCUUCAACCAGCUA
7. hsa-miR-133b UUUGGUCCCCUUCAACCAGCUA
8. mml-miR-133b-3p UUUGGUCCCCUUCAACCAGCUA
9. mmu-miR-133b-3p UUUGGUCCCCUUCAACCAGCUA
10. oan-miR-133b-3p UUUGGUCCCCUUCAACCAGCUA
11. oha-miR-133b-3p UUUGGUCCCCUUCAACCAGCUAU
12. ppy-miR-133b UUUGGUCCCCUUCAACCAGCUA
13. ptr-miR-133b UUUGGUCCCCUUCAACCAGCUA
14. rno-miR-133b-3p UUUGGUCCCCUUCAACCAGCUA
15. sme-miR-133b-3p UUGGUCCCCAUCAACCAGCA

miR-206-3p

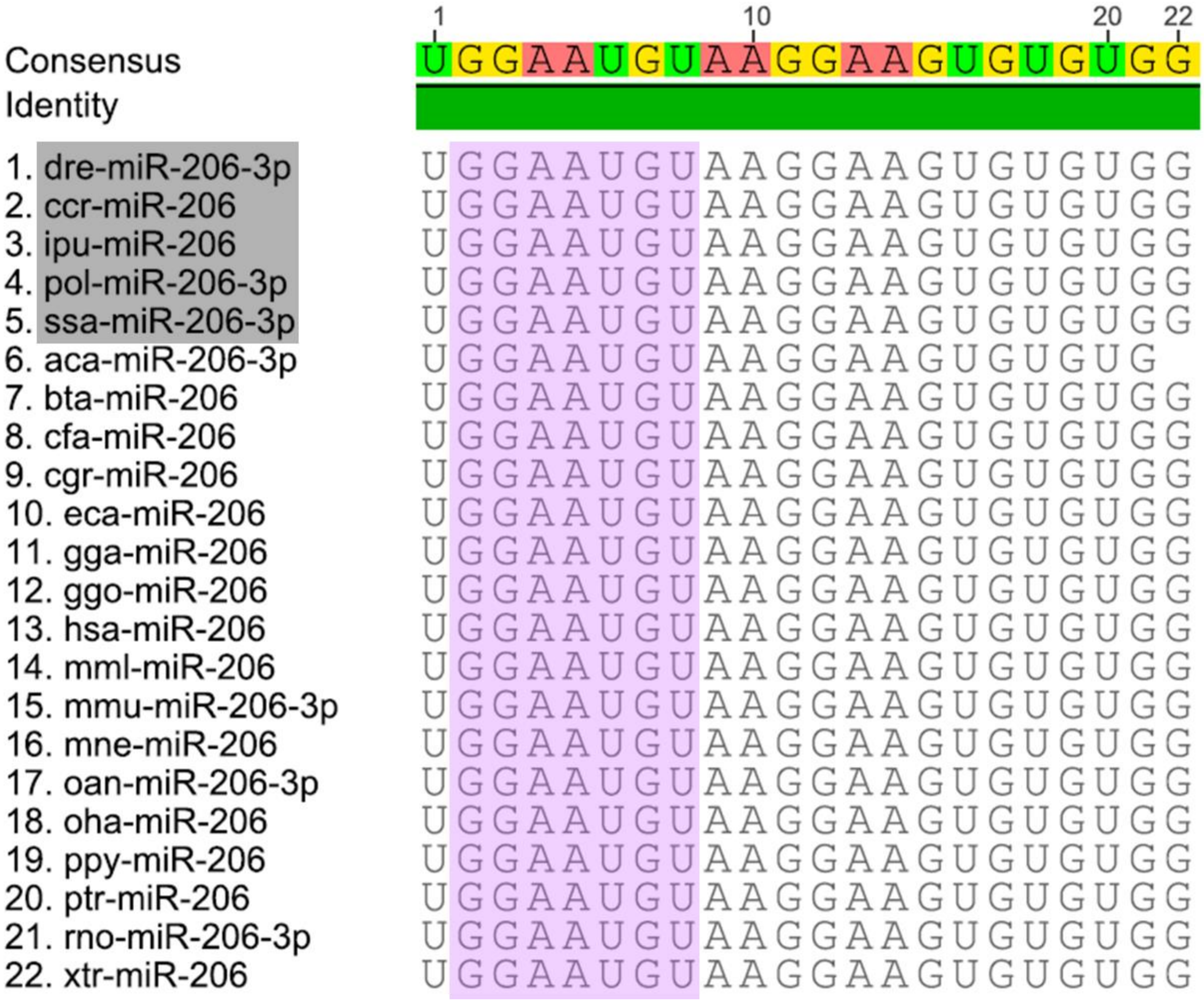

1
10
20
22
Consensus
Identity
UGGAAUGUAAGGAAGUGUGUGG
1. dre-miR-206-3p UGGAAUGUAAGGAAGUGUGUGG
2. ccr-miR-206 UGGAAUGUAAGGAAGUGUGUGG
3. ipu-miR-206 UGGAAUGUAAGGAAGUGUGUGG
4. pol-miR-206-3p UGGAAUGUAAGGAAGUGUGUGG
5. ssa-miR-206-3p UGGAAUGUAAGGAAGUGUGUGG
6. aca-miR-206-3p UGGAAUGUAAGGAAGUGUGUG
7. bta-miR-206 UGGAAUGUAAGGAAGUGUGUGG
8. cfa-miR-206 UGGAAUGUAAGGAAGUGUGUGG
9. cgr-miR-206 UGGAAUGUAAGGAAGUGUGUGG
10. eca-miR-206 UGGAAUGUAAGGAAGUGUGUGG
11. gga-miR-206 UGGAAUGUAAGGAAGUGUGUGG
12. ggo-miR-206 UGGAAUGUAAGGAAGUGUGUGG
13. hsa-miR-206 UGGAAUGUAAGGAAGUGUGUGG
14. mml-miR-206 UGGAAUGUAAGGAAGUGUGUGG
15. mmu-miR-206-3p UGGAAUGUAAGGAAGUGUGUGG
16. mne-miR-206 UGGAAUGUAAGGAAGUGUGUGG
17. oan-miR-206-3p UGGAAUGUAAGGAAGUGUGUGG
18. oha-miR-206 UGGAAUGUAAGGAAGUGUGUGG
19. ppy-miR-206 UGGAAUGUAAGGAAGUGUGUGG
20. ptr-miR-206 UGGAAUGUAAGGAAGUGUGUGG
21. rno-miR-206-3p UGGAAUGUAAGGAAGUGUGUGG
22. xtr-miR-206 UGGAAUGUAAGGAAGUGUGUGG

miR-499-5p

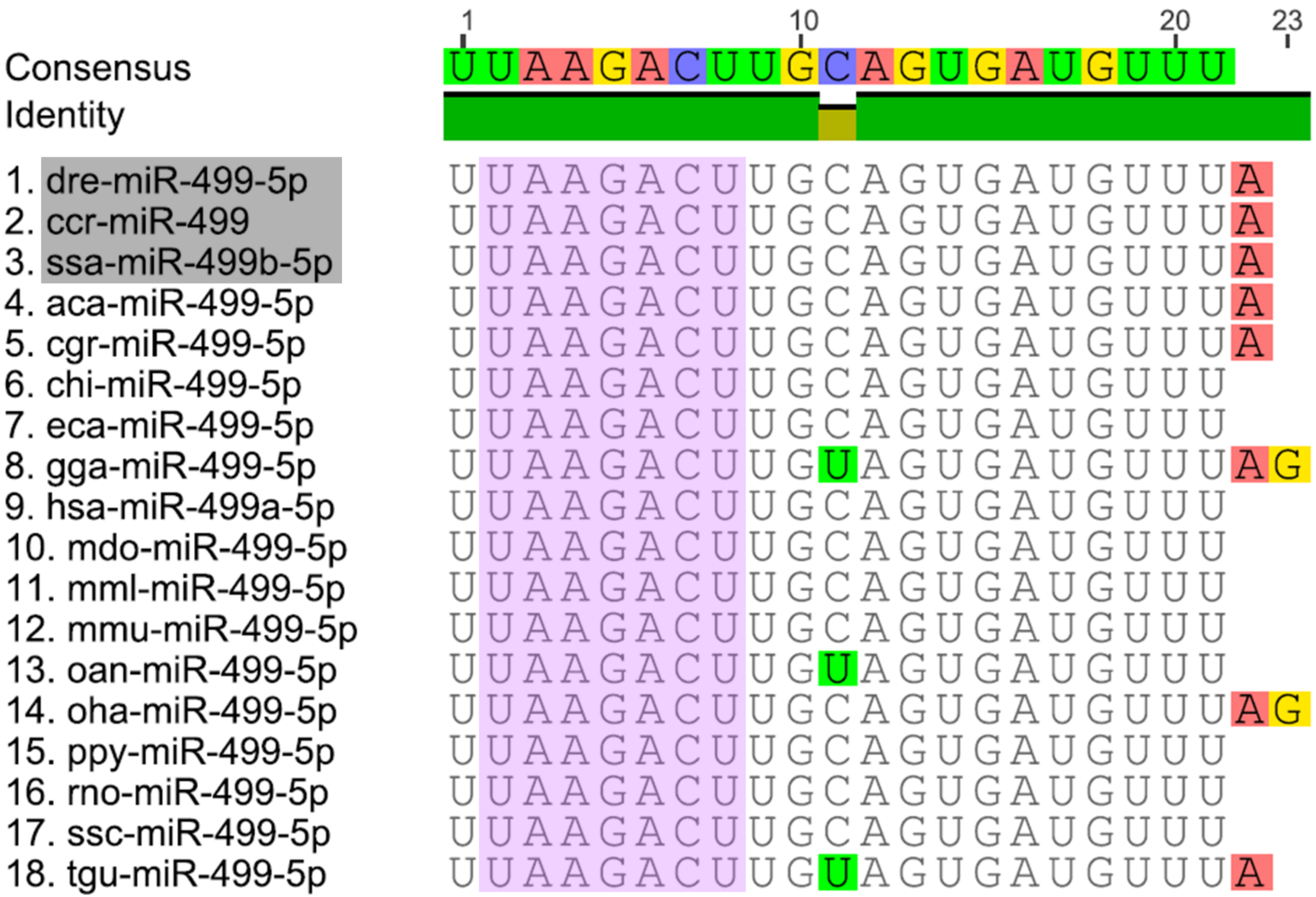

1
10
20
23
Consensus
UUAAGACUUGCAGUGAUGUUU
Identity
1. dre-miR-499-5p UUAAGACUUGCAGUGAUGUUUA
2. ccr-miR-499 UUAAGACUUGCAGUGAUGUUUA
3. ssa-miR-499b-5p UUAAGACUUGCAGUGAUGUUUA
4. aca-miR-499-5p UUAAGACUUGCAGUGAUGUUUA
5. cgr-miR-499-5p UUAAGACUUGCAGUGAUGUUUA
6. chi-miR-499-5p UUAAGACUUGCAGUGAUGUUU
7. eca-miR-499-5p UUAAGACUUGCAGUGAUGUUU
8. gga-miR-499-5p UUAAGACUUGUAGUGAUGUUUAG
9. hsa-miR-499a-5p UUAAGACUUGCAGUGAUGUUU
10. mdo-miR-499-5p UUAAGACUUGCAGUGAUGUUU
11. mml-miR-499-5p UUAAGACUUGCAGUGAUGUUU
12. mmu-miR-499-5p UUAAGACUUGCAGUGAUGUUU
13. oan-miR-499-5p UUAAGACUUGUAGUGAUGUUU
14. oha-miR-499-5p UUAAGACUUGCAGUGAUGUUUAG
15. ppy-miR-499-5p UUAAGACUUGCAGUGAUGUUU
16. rno-miR-499-5p UUAAGACUUGCAGUGAUGUUU
17. ssc-miR-499-5p UUAAGACUUGCAGUGAUGUUU
18. tgu-miR-499-5p UUAAGACUUGUAGUGAUGUUUA
