# Supplementary material for: Differential microRNA Expression in Fast- and Slow-Twitch Skeletal Muscle of Piaractus mesopotamicus during Growth
Source: PLoS One. 2015 Nov 3;10(11):e0141967. doi: 10.1371/journal.pone.0141967 (PMC4631509; doi:10.1371/journal.pone.0141967)
Supplement: S5 File — The MFE (minimum free energy) values were within accepted ranges. (PDF) [file pone.0141967.s005.pdf]

```
target: Piaractus_mesopotamicus_HDAC4_utr3
length: 712
miRNA : dre-miR-1
length: 22

mfe: -18.4 kcal/mol
p-value: 0.999931

position  366
target 5'  A  U         U   GG   C 3'
            AC   CUUCUUU CAU  CCA
            UG   GAAGAAA GUA  GGU
miRNA  3' UA  UAU       U   A      5'
```

```
target: Piaractus_mesopotamicus_SRF_3utr
length: 815
miRNA : dre-miR-133a-3p
length: 21

mfe: -21.9 kcal/mol
p-value: 0.931724

position  354
target 5' C     ACU   UU      U 3'
           GCUGG     G  GACCAA
           CGACC     C  CUGGUU
miRNA  3' U     AACUU CC      U 5'
```

```
target: Piaractus_mesopotamicus_SRF_3utr
length: 815
miRNA : dre-miR-133b-3p
length: 22

mfe: -21.9 kcal/mol
p-value: 0.949434

position  354
target 5'  C     ACU   UU      U 3'
            GCUGG     G  GACCAA
            CGACC     C  CUGGUU
miRNA  3' AU     AACUU CC      U 5'
```

```
target: Piaractus_mesopotamicus_pax7_3utr
length: 190
miRNA : dre-miR-206-3p
length: 21

mfe: -20.8 kcal/mol
p-value: 0.437849

position  36
target 5' U   UUCCCC    UC    GGGC   GUG    C 3'
           CAC      CGCU  UCCU    GCA   UCCA
           GUG      GUGA  AGGA    UGU   AGGU
miRNA  3'     U               A      A        5'
```

```
target: Piaractus_meospotmaicus_SOX6_utr_dowstream
length: 1859
miRNA : mir499
length: 22

mfe: -24.3 kcal/mol
p-value: 0.753434

position  885
target 5' A         CCUUCAGCGCUUUU        C    3'
           AAACAUCGC              UGCGGGUC
           UUUGUAGUG              ACGUUCAG
miRNA  3' A                               AAUU 5'
```
