# Supplementary material for: Differential microRNA Expression in Fast- and Slow-Twitch Skeletal Muscle of Piaractus mesopotamicus during Growth
Source: PLoS One. 2015 Nov 3;10(11):e0141967. doi: 10.1371/journal.pone.0141967 (PMC4631509; doi:10.1371/journal.pone.0141967)
Supplement: S1 Table — (PDF) [file pone.0141967.s006.pdf]

S1 Table. *TaqMan*® assays used for the miRNA and *U6 snRNA* amplification by qPCR.

miRBase ID	miRBase accession number	Mature sequence (5′ to 3′)
**dre-miR-1**	MIMAT0001768	UGGAAUGUAAAGAAGUAUGUAU
**dre-miR-133a-3p**	MIMAT0001830	UUUGGUCCCCUUCAACCAGCUG
**dre-miR-133b-3p**	MIMAT0001831	UUUGGUCCCCUUCAACCAGCUA
**dre-miR-206-3p**	MIMAT0001866	UGGAAUGUAAGGAAGUGUGUGG
**dre-miR-499-5p**	MIMAT0003749	UUAAGACUUGCAGUGAUGUUUA
U6 snRNA	NCBI Reference Sequence: NR_004394	

dre, *Danio rerio*.
