# Supplementary material for: Differential microRNA Expression in Fast- and Slow-Twitch Skeletal Muscle of Piaractus mesopotamicus during Growth
Source: PLoS One. 2015 Nov 3;10(11):e0141967. doi: 10.1371/journal.pone.0141967 (PMC4631509; doi:10.1371/journal.pone.0141967)
Supplement: S2 Table — (PDF) [file pone.0141967.s007.pdf]

S2 Table. Primers used for *hdac4, srf, pax7, sox6, myod, myogenin* and *18S* mRNA amplification by qPCR.

Gene	Forward primer (5′ to 3′)	Reverse primer (5′ to 3′)
hdac4	CCAGGACACCATGCTGAAGA	CGATGGCCACAGAGTTGAAA
srf	TCTGATAGCCAGCGTTCACTGT	TGCTCACCTGCATGGTGGTA
pax7	GTCGATGCTGTGTTTGGTCTTC	CGAGGATGAGTGCGATAAGAAA
sox6	TTCCGACGCATCAACAGTTTAA	GCAAATGACACGCCTGCAT
myod	CGCCATCAGCTACATCGAG	ATCCAGGACGGGGTAGTAGG
myogenin	TCCCAGACCAGAGGTTTTATGAA	TCTTGGTATCCTGCTTGGTCAA
18s	CGGAATGAGCGTATCCTAAACC	GCTGCTGGCACCAGACTTG
